# Mechanistic Pathways Linking Type 2 Diabetes Mellitus, Hypertension, and Osteoarthritis to Falls in Older Adults: A Scoping Review

**DOI:** 10.3390/healthcare14152271

**Published:** 2026-07-24

**Authors:** Liyang He, Siew Kuan Chua

**Affiliations:** 1 Department of Physiotherapy, School of Nursing & Applied Science, Lincoln University College, Petaling Jaya 47301, Malaysia; 2 School of Physiotherapy, Faculty of Medicine, Nursing and Health Sciences, SEGi University Kota Damansara, Petaling Jaya 47810, Malaysia; chuasiewkuan@segi.edu.my

**Keywords:** falls, older adults, type 2 diabetes mellitus, hypertension, osteoarthritis, multimorbidity, mechanisms, scoping review

## Abstract

**Background**: Falls in older adults are a major public health and rehabilitation concern and may reflect interacting chronic disease-related mechanisms rather than diagnostic labels alone. Type 2 diabetes mellitus (T2DM), hypertension (HTN), and osteoarthritis (OA) are common in later life and may influence fall vulnerability through sensory, vascular, musculoskeletal, cognitive, medication-related, and functional pathways. This scoping review aimed to map mechanism-oriented evidence linking T2DM, HTN, and OA to falls and fall-related outcomes in older adults. **Methods**: A scoping review was conducted in accordance with established scoping review methodology and PRISMA-ScR guidance. PubMed, Web of Science, and Embase were searched for English-language studies published from 2016 to 2026; the final search was conducted on 15 April 2026. Evidence was charted in a structured table and synthesised descriptively and thematically across disease-specific and shared mechanism domains. **Results**: A total of 151 studies were included. The evidence was organised into T2DM-related sensory-neural, gait and balance, vestibular/visual-cognitive, strength-related, and glycaemic or medication-related mechanisms; HTN-related haemodynamic, cerebral perfusion, dizziness/syncope, and medication-related mechanisms; and OA-related pain, proprioceptive, strength-related, gait and balance, psychological, and compensatory-control mechanisms. Shared pathways included frailty, multimorbidity, polypharmacy, pain burden, cognitive impairment, sleep disturbance, ADL decline, dizziness, and reduced physical function. **Conclusions**: Falls in older adults with T2DM, HTN, OA, or multimorbidity may be better understood through interacting disease-specific and shared mechanisms than through diagnostic labels alone. Future longitudinal and multimorbidity-specific studies using standardised fall ascertainment are needed to test these pathways more directly.

## 1. Introduction

Falls in older adults constitute a major geriatric syndrome and public health problem. The World Health Organization identifies falls as a leading cause of injury worldwide [1], and global fall-prevention guidance emphasises that fall risk is usually multifactorial, involving biological, functional, behavioural, medication-related, and environmental contributors rather than a single cause [2]. In the United States, more than one in four adults aged 65 years or older falls each year, underscoring the scale of the problem in ageing populations [3]. These events are clinically important not only because of immediate injury, but also because they may precipitate fear of falling, functional restriction, loss of independence, hospitalisation, admission to long-term care, and mortality [4,5,6,7].

T2DM [8], HTN [9], and OA [10] are highly relevant to fall risk because they are common in older adults and can affect systems required for safe standing, walking, turning, and recovery from perturbations [11]. Diabetes represents a growing global health burden; the International Diabetes Federation estimated that 589 million adults aged 20–79 years were living with diabetes in 2024, with a substantial proportion aged 65 years or older [12]. HTN is similarly prevalent, with the WHO estimating that 1.4 billion adults aged 30–79 years had hypertension in 2024 [13]. OA is a major contributor to pain and mobility limitation in older adults, and recent global burden estimates indicate that its prevalence is rising, partly because of population ageing and obesity [10].

The relationship between these chronic diseases and falls should not be interpreted as a direct effect of the diagnostic label [14]. A chronic disease diagnosis does not, in itself, cause a fall. Instead, these conditions may contribute to falls through intermediate mechanisms that impair the physiological and functional systems required for postural stability [2]. T2DM may contribute to fall vulnerability through sensory, motor, metabolic, and functional pathways, including peripheral neuropathy, impaired plantar sensation, visual impairment, cognitive slowing, vestibular dysfunction, altered foot biomechanics, gait deterioration, dynapenia, sarcopenia, and hypoglycaemia [15,16]. HTN may contribute through impaired blood-pressure regulation, orthostatic hypotension, low treated systolic blood pressure, cerebral hypoperfusion, syncope, adverse effects of antihypertensive treatment, and electrolyte disturbances [17,18,19,20,21]. OA may contribute through pain, altered joint loading, impaired proprioception, muscle weakness, reduced balance recovery, gait adaptation, concern about falling, and fear-related activity restriction [22,23,24].

This distinction is important because fall-prevention approaches based solely on diagnostic labels may overlook modifiable mechanisms [25]. These disease-specific pathways may converge on shared geriatric mechanisms such as frailty, sarcopenia, multimorbidity, polypharmacy, sleep disturbance, ADL decline, and reduced physical performance [26,27,28,29,30]. Accordingly, the same diagnostic label may represent different fall-relevant mechanisms in different older adults, whereas different diseases may converge on similar impairments in balance, gait, strength, cognition, physiological regulation, or compensatory capacity [31].

Previous reviews have addressed falls in older adults through broad risk-factor syntheses [32] or disease-specific questions concerning hypertension management [33], T2DM-related balance and fall risk [34,35], and balance or fall-related outcomes in knee OA [23]. Subsequent syntheses have largely maintained this compartmentalised focus, examining orthostatic hypotension [36], knee or hip OA [37], frailty [38], T2DM [8], or general fall-risk factors among community-dwelling older adults [39]. Although informative within their respective scopes, these syntheses have generally considered these conditions and mechanisms separately, leaving uncertainty about how T2DM-, HTN-, and OA-related pathways compare and overlap with shared geriatric and multimorbidity-related mechanisms.

The underlying primary evidence is also dispersed across several disciplines, including diabetology, cardiovascular medicine, rheumatology, geriatrics, rehabilitation, biomechanics, ophthalmology, vestibular science, pharmacology, and epidemiology. Some studies assess actual falls, whereas others use fall-related proxies such as gait speed, balance performance, postural sway, stepping accuracy, proprioception, or functional mobility. This heterogeneity supports the use of a scoping review, which is designed to map the breadth of evidence, clarify concepts, identify evidence gaps, and synthesise diverse studies without necessarily estimating a pooled effect size [40,41].

The aim of this scoping review was to identify, map, and synthesise the mechanistic pathways linking T2DM, HTN, and OA to falls and fall-related outcomes in older adults. Specifically, the review sought to organise the evidence into disease-specific and shared mechanism domains, distinguish direct fall outcomes from fall-related proxy outcomes, and develop an evidence-informed conceptual framework showing how chronic diseases may impair stability, mobility, physiological regulation, and compensatory capacity, thereby contributing to fall vulnerability.

## 2. Materials and Methods

This study was designed as a mechanism-oriented scoping review. It was informed by established scoping review methodology [41,42] and reported in accordance with PRISMA-ScR guidance [43]. The completed PRISMA-ScR checklist is provided as Supplementary File S1. The protocol was registered on the Open Science Framework (registration DOI: 10.17605/OSF.IO/X69T5). Studies were not included solely because they reported T2DM, HTN, OA, or falls as variables; they were prioritised when they contributed evidence concerning a pathway between a disease, an intermediate impairment, and a fall-related outcome.

The review question and eligibility criteria were structured using the JBI-recommended Population–Concept–Context (PCC) framework for scoping reviews [42]. The population comprised older adults or ageing populations relevant to fall risk in later life. The concept comprised mechanism-oriented evidence linking T2DM, HTN, OA, or shared geriatric or multimorbidity-related mechanisms to falls or fall-related outcomes. The context encompassed community, outpatient, hospital, long-term-care, population-based cohort, and laboratory gait or balance settings in which fall-related mechanisms could be assessed.

### 2.1. Search Strategy

The literature search covered three major international bibliographic databases: PubMed, Web of Science (WoS), and Embase. The final search was conducted on 15 April 2026. The review used 2016 as a pragmatic start date for a contemporary evidence update building on earlier general and disease-specific syntheses and was restricted to English-language publications. The strategy aimed to identify studies relevant to chronic disease-related fall mechanisms rather than studies reporting diagnostic labels only as statistical risk factors. Search concepts were organised into four blocks: falls and fall-related outcomes; older adults or ageing populations; target chronic diseases and multimorbidity-related terms; and mechanism-related concepts.

The search strategy combined controlled vocabulary, including MeSH and Emtree terms, with free-text keywords related to falls, older adults, the target chronic diseases, multimorbidity, and potential mechanistic pathways. The principal search terms included “falls”, “fall risk”, “fracture risk”, “older adults”, “elderly”, “type 2 diabetes mellitus”, “hypertension”, “osteoarthritis”, “multimorbidity”, “pathophysiology”, “mechanism”, and “risk factors”. Synonyms within each concept were combined using OR, whereas the principal concept groups were combined using AND. Truncation and database-specific controlled vocabulary were applied where appropriate. The complete search strategies for PubMed, Web of Science, and Embase are presented in Supplementary File S2.

### 2.2. Eligibility Criteria

Eligible populations comprised adults in later life, generally represented by samples aged 60 years or older, as well as ageing cohorts whose findings were relevant to fall risk in older adults. Studies involving adults with T2DM, HTN, OA, multimorbidity, frailty, sarcopenia, polypharmacy, or fall-related functional decline were eligible when their findings could inform mechanisms relevant to older adults. Eligible contexts included community, outpatient, hospital, long-term-care, population-based cohort, and laboratory settings for gait or balance assessment, provided that the study addressed fall-related mechanisms.

Eligible outcomes included actual falls, recurrent falls, injurious falls, fall-related fractures, fall-related hospitalisation, fall-related healthcare use, fall risk, fear of falling, balance, gait, mobility, postural control, and related biomechanical, physiological, cognitive, or functional proxies. Proxy outcomes were retained only when they contributed to the mechanism map and were not treated as equivalent to prospectively ascertained falls.

The core concept was the mechanism linking chronic disease to falls or fall-related outcomes. Mechanisms of interest included, but were not limited to, peripheral neuropathy, plantar sensory loss, proprioceptive impairment, visual impairment, contrast sensitivity, vestibular dysfunction, gait impairment, balance impairment, pain, muscle weakness, dynapenia, sarcopenia, orthostatic hypotension, low systolic blood pressure, cerebral hypoperfusion, syncope, blood-pressure variability, hypoglycaemia, antihypertensive medication effects, electrolyte imbalance, kinesiophobia, frailty, multimorbidity, ADL decline, sleep disturbance, and polypharmacy.

Studies were included if they addressed at least one target disease or shared geriatric pathway and contributed evidence about how the disease or condition might lead to falls or fall-related impairment. Eligible designs included observational studies (cross-sectional, longitudinal, and case–control), experimental biomechanical studies, clinical studies, and selected reviews. Primary empirical studies were prioritised. Reviews were included cautiously, mainly when they synthesised mechanisms that were not sufficiently covered by primary studies, and were treated explicitly as evidence-synthesis sources rather than primary mechanistic data.

Studies were excluded if they did not address mechanistic pathways linking T2DM, HTN, OA, or shared geriatric mechanisms to falls; were purely descriptive epidemiological studies without mechanistic interpretation; focused mainly on the consequences rather than the antecedent mechanisms of falls; lacked fall-related outcomes or relevant functional proxies; or were reviews without relevant mechanistic synthesis.

### 2.3. Study Selection

**Figure 1:**
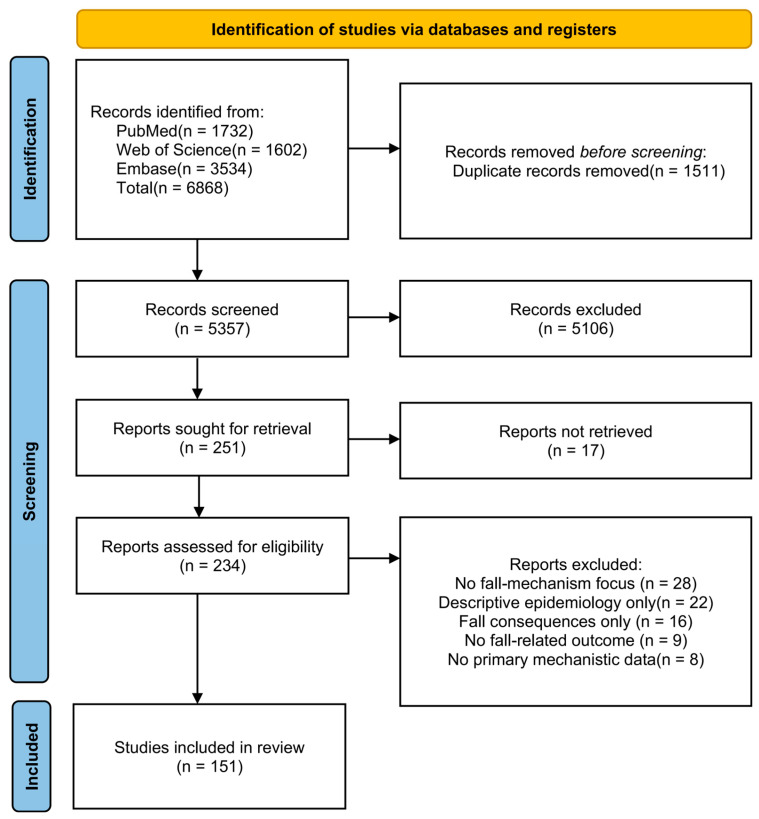
PRISMA-ScR flow diagram for study selection.

Records identified through the database searches were exported to Zotero (version 8.0.4) and imported into Rayyan (Qatar Computing Research Institute, Doha, Qatar, https://www.rayyan.ai/, accessed on 10 July 2026) for reference management and screening. After duplicate removal, two authors independently screened titles and abstracts, assessed full-text reports against the predefined eligibility criteria, and resolved disagreements through discussion and consensus. The study-selection process, including reasons for exclusion, is summarised in Figure 1.

Studies were classified separately by primary mechanism domain and primary outcome type. For primary empirical studies, the primary mechanism domain was assigned according to the principal exposure, impairment, or mechanistic pathway examined in the main analysis, with the stated objective or hypothesis used to confirm the intended focus. When these criteria diverged, priority was given to the principal factor examined in the main analysis. Primary outcome type was classified separately and did not determine mechanism-domain assignment. Review-based or contextual sources were classified according to their stated objective and principal mechanism. Studies without a predominant disease-specific mechanism were assigned to the cross-cutting or multimorbidity-related domain, while additional mechanisms were retained as secondary domains.

The evidence was synthesised descriptively and thematically. Study-selection numbers, populations, designs, outcome types, and broad mechanism categories were first summarised, after which the evidence was mapped into T2DM-related, HTN-related, OA-related, and cross-cutting or multimorbidity-related domains. Actual fall outcomes were distinguished from fall-related proxy outcomes throughout the synthesis. Meta-analysis was not conducted because the included studies were heterogeneous in design, population, mechanism measurement, and outcome ascertainment.

No formal quality assessment or risk-of-bias appraisal was conducted. This decision was consistent with the purpose of the scoping review, which was to map the breadth, characteristics, and mechanistic focus of the available evidence rather than to estimate effect sizes, grade certainty, or determine the risk of bias of individual studies. Accordingly, the included studies were not classified by quality or risk-of-bias category, and the proportion of low-quality studies was not calculated. The synthesis should therefore be interpreted as a mechanism-oriented evidence map rather than as evidence of causal strength or comparative effectiveness.

## 3. Results

### 3.1. Study Selection and Characteristics of Included Studies

The study-selection process is summarised in Figure 1. The 151 included studies spanned observational designs—including cross-sectional, cohort, retrospective, secondary-data, and case–control studies—as well as experimental or biomechanical investigations, trials, and reviews. Based on the primary mechanism-domain assignment, studies were classified as T2DM-related (*n* = 39), HTN-related (*n* = 30), OA-related (*n* = 33), or cross-cutting/multimorbidity-related (*n* = 49), and were further categorised by primary outcome type to indicate evidence directness (Table 1). Of these studies, 21 (13.9%) reported prospectively ascertained falls, whereas 48 (31.8%) reported only fall-related proxy outcomes, most commonly measures of gait, balance, mobility, postural control, or related functional, biomechanical, and physiological performance.

Supplementary File S3 summarises the bibliographic and methodological characteristics of each included study, together with participant characteristics, exposures, mechanism domains, outcome types, and main findings. Populations ranged from community-dwelling older adults and geriatric outpatients to disease-specific cohorts and broader ageing populations with multimorbidity or other geriatric vulnerabilities, such as frailty, sarcopenia, polypharmacy, pain, dizziness, and functional decline. Minimum age thresholds varied, but 60 years was used most often, and several studies enrolled clinical or disease-specific samples. Primary empirical studies informed the mapping of observed disease–mechanism–outcome relationships, whereas review-based sources provided contextual synthesis.

### 3.2. Disease-Specific and Cross-Cutting Mechanisms

#### 3.2.1. Type 2 Diabetes Mellitus-Related Mechanisms

Impairment-level evidence centred on sensory-neural and sensorimotor domains. Impaired vestibular motion perception was associated with postural instability and falls reported over one year [44]. Vestibular-specific review evidence remained incomplete and heterogeneous [45]. Broader reviews extended the focus beyond diabetic peripheral neuropathy to visual, vestibular, muscular, and cognitive abnormalities [46,47], while a review of balance problems identified sensory, motor, and cognitive contributors without establishing a single consistent impairment sequence [48]. Among proxy-focused primary studies, greater proprioceptive error and neuropathy severity were associated with poorer postural stability [49]. The musculoskeletal findings were less consistent. One review linked sarcopenia, weakness, neuropathy, and hypoglycaemia with falls or fractures [50], whereas cohort and genetic analyses linked diabetes-related foot disease to reduced strength, slower gait, and less favourable sarcopenia-related measures [51]. Among older adults with T2DM, osteoporosis was associated with dynapenia only in women [52]. Reduced muscle mass was associated with older age, lower BMI, and diabetic sensorimotor polyneuropathy in both sexes; insulin use was inversely associated with reduced muscle mass in men, whereas sulfonylurea use was positively associated in women [53].

The functional findings centred on gait, balance, mobility, and adaptive stepping. Participants with T2DM and peripheral neuropathy had poorer gait, balance, and functional performance and reported more recent falls [54]. By contrast, diabetic polyneuropathy alone did not fully distinguish fallers from non-fallers in another sample, limiting its value as an isolated discriminator of fall status in that population [55]. Neuropathy severity was associated with poorer balance and Timed Up and Go performance [56], while selected anticipatory and reactive balance deficits were also observed in participants without clinically identified neuropathy [57]. In people with diabetes, daily life and laboratory studies identified slower and less symmetrical gait, reduced visual-target stepping accuracy, and smaller adaptations in step length and minimum toe clearance [58,59,60]. Greater plantar fascia stiffness was associated with higher Falls Efficacy Scale-International (FES-I) scores and slower gait [61], while static centre-of-pressure measures indicated poorer standing control in people with T2DM [62]. Neuropathic pain was not consistently associated with additional gait or balance deficits across neuropathy groups, suggesting that pain did not uniformly add to neuropathy-related functional impairment [63].

Psychological, behavioural, cognitive, and participation-related evidence was more limited and relied predominantly on proxy outcomes. Among older adults with diabetes attending outpatient clinics, fear of falling and handgrip strength showed sex-specific associations with recorded falls [64]. Lower balance confidence and greater fear of falling were associated with fall-risk classification or T2DM status, although between-group differences in static balance remained unclear [65,66]. Among people with T2DM, coexisting vestibular dysfunction was accompanied by poorer balance, lower falls efficacy, and greater participation restriction [67]. Painful neuropathy was associated with greater kinesiophobia, lower physical activity, poorer balance, and higher fall-risk scores, but the cross-sectional design did not establish directionality [68]. For cognitive pathways, one secondary cohort analysis identified separate and serial mediation through vision and cognition between diabetes, gait impairment, and falls [69]. An experimental hyperglycaemia study subsequently identified slower attentional responses and greater centre-of-pressure travel during tandem gait, providing acute cognitive–motor evidence rather than an actual fall outcome [70]. Formal pathway testing was therefore uncommon.

Medication-related, metabolic, comorbidity-related, and fall-outcome evidence was the most heterogeneous component of the T2DM literature. Recurrent falls were associated with age, neuropathy symptoms, and self-reported balance problems [71], while possible polyneuropathy was associated with falls but not clearly with fractures [72]. Sarcopenia or dynapenia co-occurred with a higher incidence of falls [73]. Among geriatric outpatients with T2DM, fall history was associated with neuropathy, hypoglycaemia, polypharmacy, malnutrition, OA, and recent hospitalisation [74]. Among older people with diabetes receiving home nursing care, those reporting a fall during the preceding six months more frequently had impaired walking and cognitive dysfunction [75]. In a multidisciplinary fall-consultation sample, older adults with diabetes showed more cognitive decline, gait disorders, urinary incontinence, and polypharmacy than those without diabetes [76]. Frailty remained independently associated with falls after consideration of metabolic, cognitive, psychological, and functional factors [77]. In a longitudinal cohort, joint control of glycaemia, blood pressure, and lipids was not clearly associated with two-year fall risk in women, whereas glycaemic and blood-pressure control showed lower-risk associations among men [78], indicating that cardiometabolic associations were not consistent across sexes. Reviews contextualised the effects of glucose-lowering medications on bone and muscle, together with the combined contributions of neuropathy, sarcopenia, hypoglycaemia, comorbidity, and fracture vulnerability [79,80]. Related proxy-focused evidence associated neuropathy with lower femoral bone density and higher fall-risk scores [81]. Dysmobility syndrome was operationalised as the co-occurrence of at least three components selected from osteoporosis, excess adiposity, low muscle mass, low muscle strength, slow gait, and fall history, but was not itself tested as a prospective fall pathway [82].

#### 3.2.2. Hypertension-Related Mechanisms

Impairment-level HTN evidence centred on orthostatic blood-pressure (BP) regulation. Reviews described orthostatic hypotension (OH) as a heterogeneous haemodynamic disorder involving autonomic dysfunction, cerebral hypoperfusion, coexisting supine hypertension, and treatment-related BP effects [83,84,85]. Among older hypertensive outpatients, OH affected approximately one-quarter of participants and was independently associated with calcium-channel-blocker use; most participants with measured OH reported related symptoms [86]. Tilt-table data showed that supine hypertension in classical OH reflected high supine peripheral resistance and failure to augment resistance during upright posture [87]. OH was associated with lower baroreflex sensitivity and reduced recovery of cerebral oxygenation, whereas impaired cerebral autoregulation was not demonstrated, narrowing support to baroreflex and oxygenation abnormalities rather than a general autoregulatory failure [88]. During standing, hypertensive participants showed reduced premotor and supplementary-motor cortical activation despite no clear between-group difference in centre-of-pressure measures, indicating that altered cortical activation was not accompanied by measurable static postural impairment in that study [89]. An orthostatic systolic BP rise after one minute of standing, representing a different BP-response pattern, was associated with frailty, particularly reduced motor function, in older outpatients [90].

The functional and symptom-related findings were less consistent. Supine-to-standing testing identified more OH and orthostatic symptoms than seated-to-standing testing, and associations with incident falls differed between protocols, with supine systolic OH showing the clearer association [91]. Among community-dwelling older adults, substantial dizziness was associated with more falls, poorer function, lower physical activity, and poorer quality of life [92]. Vestibular syncope recurred in some patients and occasionally caused serious injury, while OH was present in only a subset, indicating that vestibular syncope and OH were not interchangeable mechanisms [93]. In a hypertensive sample, fall history was associated with limitations in basic or instrumental activities of daily living (BADL/IADL), hearing impairment, stroke, and poorer self-rated health, with several associations differing by sex [94]. Among outpatients with dizziness, hypertension was associated with abnormal posturographic balance [95].

Psychological, behavioural, sensory-processing, and neurocognitive evidence was limited. Cognitive frailty was associated with fall history in community-dwelling older adults with hypertension [96]. Among hospitalised older adults with hypertension, cognitive frailty and sleep disorders were independently associated with previous falls alongside malnutrition, polypharmacy, and the burden of coexisting chronic conditions [97]. By contrast, proxy-focused studies found poorer balance among hypertensive older women [98] and greater fear of falling and kinesiophobia, together with poorer vestibular, visual, and activity-related sensory processing, among older adults with hypertension [99].

Medication, comorbidity, and fall-outcome evidence varied according to treatment intensity, achieved BP, drug class, treatment modification, and care setting. In the Systolic Blood Pressure Intervention Trial (SPRINT), intensive treatment increased serious hypotension and possibly syncope but not serious falls, indicating that the increase in these adverse physiological events did not translate into more serious falls within the trial [19]. In a prospective cohort, treated hypertensive women had lower adjusted fall rates than normotensive women [100], whereas treated systolic BP below 110 mmHg was associated with serious falls or syncope in a different population [101]. A narrative review reported that safety findings for intensive BP control, including hypotension, syncope, and falls, varied according to frailty, the burden of coexisting chronic conditions, and outcome definition [102]. Sustained OH with coexisting hypertension was more consistently associated with future injurious or unexplained falls than OH alone [18], while pooled review evidence supported an association between OH and falls rather than a uniform effect of hypertension itself [103]. Treatment deintensification was not associated with a lower overall risk of syncope or fall-related injury [104]. Antihypertensive withdrawal showed no clear association with falls despite increasing BP and symptomatic adverse events [105]; together, these findings provided no consistent support that treatment reduction alone lowered fall-related events. Polypharmacy and diuretic use were associated with fall history, whereas the number of antihypertensive agents was not [9]. Among patients admitted with fall-related injuries, hydrochlorothiazide, ACE inhibitors, beta-blockers, and combined ACE inhibitor–hydrochlorothiazide use were more frequent in those with hyponatraemia; hyponatraemia was associated with fracture, but the absence of non-faller controls precluded estimation of fall risk [106]. An inpatient cohort documented changes in antihypertensive prescribing before and after a fall rather than estimating the effect of antihypertensive exposure on fall risk [107]. Proxy-focused evidence associated a high Downton fall-risk classification with spironolactone, beta-blocker, and calcium-channel-blocker exposure [108], while the combination of frailty and hypertension was associated with the highest prevalence of STEADI-defined fall risk [109].

#### 3.2.3. Osteoarthritis-Related Mechanisms

The OA literature, which predominantly focused on the knee, centred more consistently on symptomatic pain, sensorimotor disturbance, and reduced lower-limb capacity than on radiographic severity alone. In knee OA, pain, depression, and reduced mobility were associated with falls, whereas radiographic severity was not independently associated; functional and psychological factors partly accounted for pain–fall relationships [110,111]. These findings limited the explanatory value of radiographic severity alone and placed greater emphasis on symptomatic and functional characteristics. A review identified balance impairment, weakness, symptomatic-joint burden, and comorbidity but judged the findings for pain, instability, proprioception, and walking-aid use to be limited or conflicting [112]. Proxy-focused studies further showed that pain phenotypes were not interchangeable: greater pain sensitisation was associated with a stiffer postural strategy under sensory challenge [113], whereas neuropathic pain accompanied poorer stability and function but not a higher fall-risk classification, limiting its ability to distinguish higher classified fall risk [114]. Proprioception showed the strongest association with balance among the sensory, range-of-motion, strength, pain, and tactile measures assessed in knee OA [115]. Ankle-inversion discrimination during walking was impaired in knee OA [116].

Functional studies examined standing and walking control, stair performance, rapid stepping, and laboratory balance recovery. Fall-history-based evidence showed that fallers with hip or knee OA had posterior gait centre-of-pressure displacement and site-specific asymmetry or variability [117], while gait patterns associated with fall history differed according to knee-pain status [118]. Proxy-focused studies showed greater centre-of-pressure displacement during standing in unilateral knee OA [119] and limb-loading adaptations with greater temporal variability among women with end-stage ankle OA [120]. During stair walking, knee OA was associated with greater variability in kinematic and selected kinetic measures [121], together with altered whole-body angular momentum [122]. Stiff-knee gait was related to pain, reduced quadriceps strength, and restricted knee flexion [123], while participants with knee OA also demonstrated slower voluntary step execution [124]. Simulated or induced-fall tasks revealed altered recovery kinematics and poorer stability responses [125,126]. However, another study found OA-related differences after laboratory-induced trips but not across the principal kinematic measures following treadmill perturbations, and experimental fall occurrence did not differ significantly by OA status; this limited the consistency of perturbation-based findings across testing paradigms [127].

Psychological and behavioural evidence was more limited. A prospective cohort provided the most direct pathway evidence: concern about falling, knee-extension torque, and postural sway partly mediated the association between knee pain and multiple falls [128]. A case–control study also identified fear of falling and depressive symptoms within the association between OA symptom severity and previous falls, although the design could not establish temporal direction [129]. Across knee and hip OA samples, greater pain, poorer physical function, fear of movement, impaired balance, and lower fall self-efficacy were interrelated [130,131,132].

OA fall-outcome evidence remained heterogeneous in its definitions of OA, pain exposures, and fall ascertainment. Prospective evidence was concentrated mainly in knee OA. Population-based follow-up identified knee OA as an independent correlate of future injurious falls [133], while longitudinal Osteoarthritis Initiative data associated radiographic knee OA with recurrent falls [134]. In a separate Osteoarthritis Initiative analysis, multimorbidity coexisting with knee OA was associated with a greater number of falls over follow-up but not with a statistically significant increase in the likelihood of reporting any fall, suggesting a closer relationship with fall burden than with the occurrence of at least one fall [135]. In a cross-sectional knee OA sample, proprioception, range of motion, symptoms, fear of falling, low back pain, diabetes, and body mass index were associated with previous falls [22]. Hip evidence was more limited: a greater burden of hip OA signs and symptoms predicted falls over one year among adults with chronic low back pain [136], whereas women with end-stage hip OA reported more previous falls, injuries, and fractures, with limping and knee-extensor weakness distinguishing fallers [137]. Moderate-to-severe low back pain and multisite pain were associated with recurrent or incident falls [138,139]. Falls were more prevalent in generalised than in localised OA; in adjusted analyses, hypertension and antidepressant use were associated with any fall, whereas hypertension, neuropathy, and insulin use were associated with recurrent falls [140]. Opioid and antidepressant use predicted subsequent recurrent falls after adjustment for pain and depression severity, whereas associations differed across other analgesic categories [141].

#### 3.2.4. Cross-Cutting and Multimorbidity-Related Mechanisms

##### Multimorbidity and Cumulative Disease Burden

Across the included studies, multimorbidity was operationalised as either the number of coexisting chronic conditions or recurring multimorbidity patterns. In a three-year cohort, each additional condition and baseline multimorbidity predicted higher rates of all and injurious falls [142]. The presence of at least three conditions was also associated with falls and fall-related injury [143], while other studies associated greater chronic disease burden with falls or recurrent falls [144,145,146]. Higher chronic disease counts were associated with recurrent falls in both younger-old and older-old adults [147]. Associations varied by disease composition and fall outcome. Multimorbidity was more strongly associated with frequent nonslip than slip falls [148], while cardiac-metabolic, visceral-arthritic, respiratory, and mental-sensory patterns were associated with higher fall risk [149]. Fall-related injury increased with the number of physical conditions and was independently associated with arthritis, visual difficulty, chronic lung disease, chronic back pain, hearing problems, and stroke [150]. Cardiometabolic and mental-health/musculoskeletal patterns were associated with fall-related hospitalisation, with the cardiometabolic pattern also associated with fracture-related hospitalisation [151].

##### Frailty, Sarcopenia or Dynapenia, and Reduced Functional Reserve

Frailty trajectories [152] and severe sarcopenia [153] were associated with falls, whereas dynapenia was associated with fall history, slower gait, and ADL limitation [154]. In a cohort of community-dwelling older adults, self-reported physical function was more consistently associated with subsequent falls than performance-based measures [155]. Reduced functional reserve was also reflected in multimorbidity-pattern analyses: degenerative-disease and stroke–respiratory–depression patterns were associated with poorer gait, balance, and lower-extremity muscle function [29]. A cross-sectional study treated gait stability and variability as fall-related functional proxies [156].

##### Polypharmacy and Medication-Related Mechanisms

The findings varied by drug class, exposure definition, and care setting. Medication count was associated with both gait performance and falls [157]. Drug-specific evidence associated opioid use, anticholinergic burden, and potentially inappropriate prescribing with falls in community and hospital settings [158,159,160,161,162,163]. However, a systematic review of older adults with T2DM found no clear association between polypharmacy and falls or fall risk among the included fall-related studies, limiting the interpretation of medication count alone as a uniform fall-related mechanism in this population [164]. Proxy-focused studies associated polypharmacy with poorer postural stability [165], cardiac autonomic neuropathy [166], and slower gait [167]; among older adults exposed to polypharmacy, participants with a fall history also showed poorer balance, greater kinesiophobia, and slower mobility [168]. Excessive polypharmacy was associated with a greater number of medications classified as linked to geriatric syndromes, including falls, urinary incontinence, and depression [169].

##### Pain-Related and Psychological–Behavioural Mechanisms

Direct fall evidence associated pain with fall history, recurrent falls, or injurious falls [170,171,172]. Pain intensity was associated with recurrent falls among adults aged 65–79 years but not among those aged 80 years or older, possibly because of limited statistical power [173]. Multisite pain predicted self-reported falls and falls requiring primary healthcare but not falls requiring secondary-care admission, indicating that the association differed by outcome source and severity [174]. The review findings were mixed: one review supported an association between pain and poorer balance, whereas another found slower gait but inconsistent differences in other gait characteristics [175,176]. Proxy-focused studies associated pain intensity, persistence, or distribution with fall-risk profiles or reactive balance [177,178]. Cardiopulmonary, musculoskeletal, and vascular–metabolic multimorbidity patterns were associated with fear of falling, with the strongest association observed among older adults with two or three coexisting patterns [179]. In a geriatric outpatient sample, kinesiophobia was strongly correlated with fear of falling, although the cross-sectional design did not establish temporal direction [180]. Selective attention partly mediated pain–gait associations in another study [181].

##### Sleep, Cognitive, Sensory, and Vestibular Mechanisms

Evidence concerning sleep, cognitive, sensory, and vestibular mechanisms was limited and heterogeneous. Short sleep duration of less than 6.5 h was longitudinally associated with incident falls; impaired physical function, greater chronic disease burden, poorer cognitive function, and IADL dependence partly mediated this association [182]. Very short and long sleep durations were cross-sectionally associated with fall-related fractures [183]. Progressive or persistent peripheral sensory loss was associated with gait decline and falls [184], whereas visual impairment was associated with falls or multiple falls [185,186]. A retrospective study reported a reduction in fall frequency following management of benign paroxysmal positional vertigo [187]. A systematic review linked vestibular dysfunction with falls and poorer walking function [188]. A broader review provided contextual support for the interaction of vestibular dysfunction with other age-related contributors to balance and gait disorders [31].

## 4. Discussion

### 4.1. Summary of Principal Findings

This scoping review provides a comparative synthesis of how T2DM, HTN, and OA may contribute to fall vulnerability through distinct but overlapping mechanisms. Evidence related to T2DM centred mainly on sensory-neural, sensorimotor, and gait–balance impairments, whereas HTN evidence focused on haemodynamic dysregulation, dizziness or syncope, and treatment-related contexts, and OA evidence on pain, sensorimotor dysfunction, muscle weakness, and biomechanical adaptation. Across these domains, recurring functional consequences included impairments in balance, gait, mobility, postural transitions, and compensatory control. The principal finding is therefore that fall vulnerability may reflect the cumulative disruption of sensory integration, haemodynamic regulation, musculoskeletal capacity, and adaptive reserve rather than a direct effect of diagnosis alone. This interpretation may explain why different diseases produce similar functional limitations, while people with the same diagnosis present with different fall-related profiles.

This interpretation should be considered alongside substantial within-disease heterogeneity. In T2DM, susceptibility-associated genetic variation provides contextual support for biologically distinct profiles within the same diagnostic category [189,190,191,192]. HTN is similarly a multifactorial condition arising from interactions among renal, vascular, neurohumoral, genetic, and environmental factors [193,194], whereas OA is a heterogeneous whole-joint disease characterised by diverse biomechanical, inflammatory, metabolic, and molecular processes [195,196,197]. Diagnostic labels therefore do not define a single pathophysiological route or a uniform pattern of fall-related impairment. Cross-cutting factors, including multimorbidity, frailty, pain, cognitive impairment, and medication burden, may operate across these pathways by modifying compensatory capacity or contributing to accumulated vulnerability.

Few studies simultaneously assessed disease or treatment exposure, intermediate impairment, functional limitation, and subsequent falls within a single longitudinal analysis. Three disease-specific questions therefore remain unresolved. In T2DM, despite consistent associations between DPN and functional impairment, it remains unclear whether DPN sufficiently accounts for fall vulnerability or whether other sensory, cognitive, muscular, and metabolic pathways contribute independently. In HTN, uncertainty centres on whether fall vulnerability arises from antihypertensive treatment itself or from particular haemodynamic states and treatment contexts. In OA, it remains unresolved whether symptomatic and functional characteristics explain fall vulnerability more effectively than radiographic severity. Evidence directness also differed: prospective fall ascertainment was limited in T2DM, HTN findings were largely context-specific, and OA studies more often examined prospective falls or injuries but remained predominantly knee-focused and frequently proxy-based. These differences likely reflect variation in study design and research emphasis rather than differences in clinical relevance or causal strength.

### 4.2. Integrated Mechanism-Oriented Model

**Figure 2:**
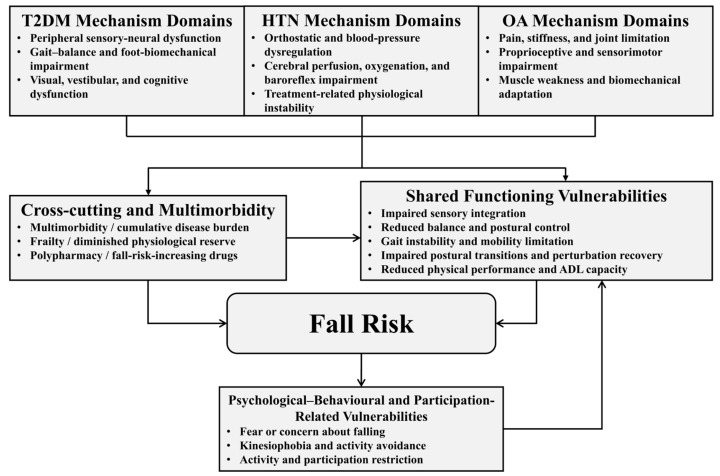
Evidence-informed conceptual framework of disease-specific, shared, and cross-cutting mechanisms related to fall risk in older adults.

The findings can be interpreted within an International Classification of Functioning, Disability and Health (ICF)-informed framework in which T2DM, HTN, and OA represent health-condition entry points rather than fixed stages in a disease-to-fall sequence [198]. Figure 2 distinguishes disease-specific pathways from the shared functional vulnerabilities through which they may influence fall risk. It also recognises that geriatric, comorbidity-related, and treatment-related influences may operate across several levels of the framework rather than occupying a single position.

At the level of body functions and structures, the three diseases showed distinct mechanistic emphases but were not functionally isolated. T2DM-related sensory-neural impairment, HTN-related haemodynamic dysregulation, and OA-related pain–sensorimotor dysfunction represent distinct routes by which sensory integration, postural regulation, lower-limb control, or physiological reserve may be compromised. Their importance within the model lies less in the diagnostic category itself than in whether the resulting impairment disrupts the capacities required for stable movement and adaptation.

At the activity level, these disease-specific disturbances converged most clearly on limitations in gait, balance, mobility, postural transitions, and recovery from perturbations. The evidence supporting impairment–function associations was more consistently represented than evidence supporting complete pathways to subsequent falls. Functional, biomechanical, physiological, and laboratory measures helped identify where mobility control may be disrupted, but did not provide direct evidence of prospectively ascertained falls. In most cases, the full sequence from disease or treatment exposure to impairment, functional limitation, and fall outcome was assembled across separate studies rather than tested within a single longitudinal analysis.

Psychological and behavioural responses and participation restrictions may interact with these functional changes rather than occurring solely as downstream consequences. Pain or fear of falling may reduce confidence, restrict activity, and contribute to declining strength or reserve, while functional deterioration or a previous fall may further reinforce fear, altered movement patterns, and participation restriction. The selected mediation findings support parts of these relationships, but their direction and timing remain incompletely established.

Cross-cutting influences are therefore represented across the framework rather than as an additional step [199]. Multimorbidity, frailty, sarcopenia, pain burden, cognitive or sleep impairment, and medication exposure may modify the expression of disease-specific impairments, reduce compensatory capacity, or reflect accumulated vulnerability. Depending on the study context, they may function as modifiers, possible mediators, confounders, vulnerability markers, or consequences of interacting conditions; the partial and variable mediation reported for activity limitation, pain, and medication-related factors is consistent with this interpretation. The connections in Figure 2 are context-dependent, vary in evidential directness, and may be bidirectional. Accordingly, the framework should not be interpreted as a validated causal model or as representing a uniform disease-to-fall sequence.

### 4.3. Implications for Assessment and Fall-Prevention Practice

The findings may inform assessment and fall-prevention practice; however, this review did not evaluate intervention effectiveness, validate a mechanism-oriented classification, or establish a required assessment pathway. Its practical value lies in helping healthcare professionals relate the chronic disease context to the way fall vulnerability is expressed in an individual.

A diagnosis or an isolated abnormal test result should not be assumed to explain why a person falls. Its relevance is more likely to depend on whether it corresponds with the circumstances of previous falls, difficulties encountered during everyday mobility, and recent changes in health or treatment. Assessment findings may therefore be more informative when interpreted as part of the individual’s overall presentation rather than as independent risk markers. This interpretation may help distinguish abnormalities that are merely present from those that plausibly contribute to current instability.

The review also highlights the potential value of integrating rehabilitation findings with the person’s broader medical and treatment context. Movement and functional difficulties may be interpreted differently when considered alongside disease stability, general health status, and medication exposure. Such integration could complement established individualised multifactorial fall assessment by reducing fragmentation between professional and disease-specific perspectives [200,201]. Reassessment may also be appropriate when changes in health, treatment, activity, or fall history alter the clinical presentation.

For fall-prevention planning, the mapped evidence may help identify potentially modifiable problems that closely correspond to the person’s current functional difficulties. Used in this way, the synthesis may support clinical reasoning and help determine which problems warrant closer assessment, monitoring, or intervention within established multifactorial care.

### 4.4. Strengths, Limitations, and Future Directions

This review has several strengths. Its mechanism-oriented scoping design, explicit primary-domain assignment, and structured data-charting process enabled heterogeneous evidence across T2DM, HTN, OA, and shared geriatric mechanisms to be organised within an integrated mechanism-domain map.

Several limitations should be acknowledged. No formal quality assessment, risk-of-bias appraisal, or certainty-of-evidence assessment was undertaken. The evidence base relied heavily on cross-sectional, retrospective, secondary-data, and laboratory studies. Heterogeneity in study design, outcome ascertainment, and mechanism-domain classification, together with unavoidable overlap across domains, precluded meta-analysis. The search was limited to PubMed, Web of Science, and Embase, restricted to English-language publications from 2016 to 2026, and did not systematically include grey literature. Pre-2016 studies were used only for contextual interpretation and were not systematically screened or charted; these restrictions may have introduced publication, language, and temporal bias.

Future studies should prospectively test complete disease–impairment–function–fall pathways and characterise the specific muscles, joints, and other tissues that contribute to, or are affected by, functional impairment. Such studies should use standardised fall ascertainment and formal mediation or interaction analyses, particularly in T2DM and HTN, for which direct prospective evidence remains limited. Applying comparable methods across T2DM, HTN, OA, and multimorbidity would help empirically evaluate the proposed framework and clarify the roles of cross-cutting factors within these pathways.

## 5. Conclusions

This scoping review mapped 151 studies and identified evidence that T2DM, HTN, OA, and multimorbidity may contribute to falls and fall-related outcomes through disease-specific and shared mechanisms rather than through diagnostic labels alone. These mechanisms involve sensory, motor, vascular, musculoskeletal, cognitive, psychological, medication-related, and broader geriatric systems required for standing, walking, turning, obstacle negotiation, and recovery from perturbations. Because many included studies assessed fall-related proxy outcomes, the mapped pathways should be interpreted as plausible rather than confirmed causal sequences. Future research should use longitudinal designs, standardised fall definitions, prospective fall ascertainment, mediation or pathway analyses, and multimorbidity-specific designs to test these mechanisms more directly.

## Figures and Tables

**Table 1:** Distribution of included studies by disease/mechanism domain and primary outcome type.

Disease/ Mechanism Domain	Prospective Fall Outcomes	Retrospective Fall Outcomes/ Fall History	Injurious Falls/ Fractures/ Healthcare Use	Proxy-Only Outcomes	Review/ Contextual Sources	Total Studies
T2DM-related mechanisms	1	13	1	17	7	39
HTN-related mechanisms	3	8	6	8	5	30
OA-related mechanisms	6	7	3	16	1	33
Cross-cutting/multimorbidity-related mechanisms	11	15	11	7	5	49
Total	21	43	21	48	18	151

## Data Availability

No new data were created or analyzed in this study. Data sharing is not applicable to this article.

## Supplementary Materials

The following supporting information can be downloaded at https://www.mdpi.com/article/10.3390/healthcare14152271/s1, Supplementary File S1: PRISMA-ScR Fillable Checklist; Supplementary File S2: Search Strategies for PubMed, Web of Science, and Embase; Supplementary File S3: Characteristics of the Included Studies.
