# Supplementary material for: Mechanistic Pathways Linking Type 2 Diabetes Mellitus, Hypertension, and Osteoarthritis to Falls in Older Adults: A Scoping Review
Source: Healthcare (Basel). 2026 Jul 24;14(15):2271. doi: 10.3390/healthcare14152271 (PMC13465934; doi:10.3390/healthcare14152271)
Supplement: Supplementary file 1 [file healthcare-14-02271-s001.zip › S2 Search strategies from each database.pdf]

Search strategies from each database.

Database	Search Strategy/Terms	Results	Date
PubMed	(("Accidental Falls"[MeSH] OR "Accidental Falls"[Title/Abstract] OR "Fall*"[Title/Abstract] OR "Falls*"[Title/Abstract] OR "Fall Risk*"[Title/Abstract] OR "Fracture Risk*"[Title/Abstract]))<br>AND (("Aged"[MeSH] OR "Aged"[Title/Abstract] OR "Older Adult*"[Title/Abstract] OR "Elderly"[Title/Abstract] OR "Geriatrics"[MeSH] OR "Geriatrics"[Title/Abstract] OR "Gerontology"[Title/Abstract] OR "Aging Population"[Title/Abstract] OR "Older People"[Title/Abstract] OR "Senior*"[Title/Abstract]))<br>AND (("Hypertension"[MeSH] OR "High Blood Pressure"[Title/Abstract] OR "HTN"[Title/Abstract] OR "Diabetes Mellitus, Type 2"[MeSH] OR "Type 2 Diabetes"[Title/Abstract] OR "Diabetes"[Title/Abstract] OR "T2DM"[Title/Abstract] OR "NIDDM"[Title/Abstract] OR "Osteoarthritis"[MeSH] OR "Osteoarthrosis"[Title/Abstract] OR "Degenerative Joint Disease"[Title/Abstract] OR "Degenerative Arthritis"[Title/Abstract] OR "Multimorbidity"[MeSH] OR "Multiple chronic conditions"[Title/Abstract] OR "Metabolic Syndrome"[MeSH] OR "Chronic Disease*"[Title/Abstract] OR "Comorbidity"[Title/Abstract] OR "Polypathology"[Title/Abstract] OR "Syndemic"[Title/Abstract]))<br>AND (("Risk Factor*"[Title/Abstract] OR "Pathophysiology"[Title/Abstract] OR "Pathology"[MeSH] OR "Pathology"[Title/Abstract] OR "mechanism*"[Title/Abstract] OR "pathogenesis"[Title/Abstract] OR "etiology"[Title/Abstract] OR "physiologic processes"[Title/Abstract] OR "biological markers"[Title/Abstract] OR "age-related changes"[Title/Abstract] OR "inflammation"[Title/Abstract] OR "senescence"[Title/Abstract] OR "degeneration"[Title/Abstract] OR "biomechanic*"[Title/Abstract] OR "neurophysiological"[Title/Abstract] OR "musculoskeletal physiological phenomena"[MeSH]))	1732	15/April /2026
Web of Science	TS=(("Accidental Fall*" OR "Fall*" OR "Fall Risk*" OR "Fracture Risk*") AND ("Aged" OR "Older Adult*" OR "Elderly" OR "Geriatric*" OR "Gerontology" OR "Aging Population" OR "Older People" OR "Senior*") AND ("Hypertension" OR "High Blood Pressure" OR "HTN" OR "Diabetes Mellitus" OR "Type 2 Diabetes" OR "T2DM" OR "NIDDM" OR "Osteoarthritis" OR "Osteoarthrosis" OR "Degenerative Joint Disease" OR "Degenerative Arthritis" OR "Multimorbidity" OR "Multiple chronic conditions" OR "Metabolic Syndrome" OR "Chronic Disease*" OR "Comorbidity" OR "Polypathology" OR "Syndemic") AND ("Risk Factor*" OR "Pathophysiology" OR "Pathology" OR "mechanism*" OR "pathogenesis" OR "etiology" OR "physiologic processes" OR "biological markers" OR "age-related changes" OR "inflammation" OR "senescence" OR "degeneration" OR "biomechanic*" OR "neurophysiological"))	1602	15/April /2026
Embase	('fall'/exp OR 'falling'/exp OR 'fall risk':ab,ti OR 'postural balance':ab,ti OR 'fracture risk':ab,ti) AND ('aged'/exp OR 'elderly':ab,ti OR 'older adult*':ab,ti OR 'geriatrics'/exp OR 'gerontology':ab,ti OR 'aging population':ab,ti OR 'senior*':ab,ti) AND ('hypertension'/exp OR 'high blood pressure':ab,ti OR 'diabetes mellitus'/exp OR 'type 2 diabetes':ab,ti OR 't2dm':ab,ti OR 'osteoarthritis'/exp OR 'osteoarthrosis':ab,ti OR 'degenerative joint disease':ab,ti OR 'multimorbidity'/exp OR 'multiple chronic conditions':ab,ti OR 'metabolic syndrome'/exp OR 'chronic disease*':ab,ti OR 'comorbidity'/exp OR 'syndemic':ab,ti) AND ('pathophysiology'/exp OR 'pathology'/exp OR 'mechanism*':ab,ti OR 'pathogenesis':ab,ti OR 'etiology':ab,ti OR 'biological marker':ab,ti OR 'inflammation'/exp OR 'senescence':ab,ti OR 'degeneration':ab,ti OR 'biomechanic*':ab,ti OR 'risk factor':ab,ti)	3534	15/April /2026
