# Supplementary material for: Mechanistic Pathways Linking Type 2 Diabetes Mellitus, Hypertension, and Osteoarthritis to Falls in Older Adults: A Scoping Review
Source: Healthcare (Basel). 2026 Jul 24;14(15):2271. doi: 10.3390/healthcare14152271 (PMC13465934; doi:10.3390/healthcare14152271)
Supplement: Supplementary file 1 [file healthcare-14-02271-s001.zip › S3 Characteristics of the Included studies.pdf]

Characteristics of the Included studies

Author, year	Location / data source	Study design	Objective	Sample size	Age	Sex / gender	Disease category and mechanism domain	Outcome measures	Main results
Nugraha, 2024	Indonesia; first-author affiliation	Literature review	To describe risk factors, balance assessment tools, and nonpharmacological management for balance disorders in older adults with diabetes mellitus.	N/A; literature review	Older adults; exact age NR	N/A; review	T2DM-related mechanisms — Balance dysfunction; neuropathy / sensory-feedback failure; cognitive impairment; gait impairment	Balance performance; fear of falling; Berg Balance Scale; Mini-BESTest; center of pressure; Timed Up and Go; diabetic peripheral neuropathy	The review identified diabetic peripheral neuropathy, sensory decline, motor impairment, and cognitive decline as risk factors for balance problems in older adults with diabetes and summarized commonly used balance assessment and exercise-based management approaches.
Han et al., 2023	United Kingdom / English Longitudinal Study of Ageing (ELSA), wave 9	Secondary analysis of cohort data	To examine whether vision and cognition serially mediated the associations of diabetes mellitus with gait speed impairment and falls in older adults.	n = 5,496	≥60 years	Female n = 3,037 (55.26%); male n = 2,459 (44.74%)	T2DM-related mechanisms — Vision impairment / visual-cognitive mediation; cognitive impairment; gait impairment	Falls; impaired gait speed; vision; cognition	Diabetes was associated with impaired gait speed and falls. Vision and cognition each mediated these associations, with smaller serial mediation through vision then cognition.
Sarodnik et al., 2018	Netherlands; first-author affiliation	Narrative review	To review fracture risk in type 2 diabetes mellitus with emphasis on sarcopenia, falls, and related mechanisms.	N/A; narrative review	NR	N/A; review	T2DM-related mechanisms — Sarcopenia / dynapenia; gait impairment; neuropathy / sensory-feedback failure	Falls; fall risk; fractures; sarcopenia; muscle strength; diabetic peripheral neuropathy; medication exposure	The review summarized mechanisms by which T2DM may increase falls and fracture risk, including reduced muscle strength/function, sarcopenia, neuropathy, hypoglycemia, retinopathy, and medication-related factors.
Aslibeigi et al., 2022	Iran / Kermanshah University of Medical Sciences	Cross-sectional study	To compare fear of falling, balance confidence, mobility, and static balance between older adults with type 2 diabetes and healthy controls.	n = 122; T2DM n = 63 and control n = 59	T2DM group 65.94 ± 4.80 years; control group 65.72 ± 4.66 years	T2DM group: female 51.92%, male 48.07%; control group: female 44.06%, male 55.93%	T2DM-related mechanisms — Balance dysfunction; gait impairment; fear of falling; multiple diabetes-related mechanisms	Fear of falling; Activities-specific Balance Confidence Scale; Timed Up and Go; modified Romberg test; balance confidence	Older adults with T2DM had higher fear of falling, lower balance confidence, and slower Timed Up and Go performance than controls. Static balance differences were not clearly significant.
Yin et al., 2026	United States / NHANES; China / CHARLS; FinnGen GWAS datasets	Cross-sectional and longitudinal cohort analyses plus Mendelian randomization	To investigate whether diabetes-related foot disease was associated with increased risk of sarcopenia-related traits.	n = 48,831 cross-sectional participants; CHARLS follow-up n = 9,093 without baseline SRTs	NHANES/CHARLS adult and older samples; exact pooled age NR	NR	T2DM-related mechanisms — Foot complications; neuropathy / sensory-feedback failure; sarcopenia / dynapenia; gait impairment	Sarcopenia-related traits; SARC-F; SPPB; grip strength; walking speed; falls score; diabetic neuropathy; diabetic foot disease	Diabetes-related foot disease was associated with poorer sarcopenia-related traits, including SARC-F, SPPB, grip strength, and falls score. Genetic analyses supported neuropathic and circulatory contributions to weaker strength and slower gait.
Suda et al., 2026	Brazil / community-dwelling people with diabetes and DPN	Cross-sectional retrospective study	To identify diabetes-related predictors of recurrent falls among community-dwelling people with diabetes and diabetic peripheral neuropathy.	Original dataset: non-fallers n = 534, fallers n = 101; modeling sample: training n = 166, test n = 101	Community-dwelling adults with diabetes; fallers 65.5 ± 9.9 years, non-fallers 61.2 ± 11.0 years in training sample	Training sample: non-fallers 58.8% male; fallers 37.8% male	T2DM-related mechanisms — Neuropathy / sensory-feedback failure; balance dysfunction; recurrent falls	Recurrent falls; MNSI; diabetic peripheral neuropathy severity; balance problems; visual deficiency; gait aid use	Age, neuropathy symptom score, and self-reported balance problems predicted recurrent falls. A second model also identified DPN severity as a fall predictor.
Hewston & Deshpande, 2016	Canada; first-author affiliation	Narrative review	To review diabetes-related sensorimotor and cognitive mechanisms that may contribute to balance impairment and fall risk beyond diabetic peripheral neuropathy.	N/A; narrative mini-review	Older adults; exact age NR	N/A; narrative review	T2DM-related mechanisms — multiple diabetes-related mechanisms; sensory impairment; vestibular dysfunction; cognitive impairment; muscle dysfunction	Falls; fall risk; balance impairment; diabetic peripheral neuropathy; sensory function; vestibular function; visual function; muscle function; cognitive function	The review summarized that T2DM-related fall risk may involve multiple mechanisms beyond DPN, including subtle sensory, vestibular, muscular, and executive-function deficits that can impair balance.
Dinh et al., 2025	Vietnam / national geriatric hospital and provincial geriatric departments	Cross-sectional study	To examine the association between frailty and fall risk among older Vietnamese outpatients with type 2 diabetes.	n = 740	≥60 years	Female 63.9%	T2DM-related mechanisms — Frailty; sarcopenia; cognitive impairment; depression; multiple diabetes-related mechanisms	Falls; recurrent falls; frailty; Clinical Frailty Scale; sarcopenia; cognitive impairment; depression; urinary incontinence; glycemic control	Frailty was strongly associated with falls among older adults with T2DM after multivariable adjustment. Advanced age, poor glycemic control, diabetic complications, depression, urinary incontinence, cognitive impairment, and sarcopenia were additional fall-related factors.
AL-Musawe et al., 2019	Portugal; first-author affiliation	Systematic review and meta-analysis	To summarize evidence on polypharmacy and adverse health consequences among elderly people with type 2 diabetes mellitus.	16 studies; meta-analysis used 3 studies	≥65 years	N/A; systematic review did not report participant-level sex/gender	Shared / multimorbidity-related mechanisms — Polypharmacy / fall-risk-increasing drugs; multimorbidity	Polypharmacy; falls; hospitalization; all-cause mortality; myocardial infarction; stroke; drug-drug interactions	Polypharmacy was associated with increased all-cause mortality and myocardial infarction in pooled analyses, while associations with stroke and hospitalization were not statistically significant. The review reported no association between fall or fall risk and polypharmacy in the fall-related included study.
Hulshof et al., 2024	Netherlands / DIALOAD and DWELL-NL studies	Cross-sectional study	To examine whether peripheral neuropathy was associated with daily-life gait quality characteristics in people with diabetes.	n = 98; peripheral neuropathy n = 68	Mean 68 ± 7 years	Female n = 32; male n = 66	T2DM-related mechanisms — Neuropathy / sensory-feedback failure; gait impairment	Daily-life gait quality; walking speed; gait intensity; gait symmetry; gait variability; peripheral neuropathy; SPPB	Peripheral neuropathy was associated with slower daily-life walking speed and poorer gait intensity and symmetry characteristics. These gait-quality impairments may indicate increased fall risk in people with diabetes.
Martin et al., 2023	Australia / Victoria University laboratory setting	Experimental / biomechanical laboratory study	To investigate the effect of diabetes mellitus on step length and minimum toe clearance adaptation using a non-immersive virtual-reality system.	n = 48; young adults n = 16, healthy older adults n = 16, older adults with diabetes n = 16	Young adults 26 ± 5 years; healthy older adults 68 ± 5 years; diabetes group 70 ± 5 years	Young adults female n = 7; healthy older adults female n = 6; diabetes group female n = 6	T2DM-related mechanisms — Gait impairment; foot clearance; adaptive gait control	Step length; minimum toe clearance; adaptive gait; treadmill walking; visual targets; gait error	Older adults with diabetes had reduced step-length and minimum-toe-clearance adjustments, with greater target-matching errors during adaptive treadmill walking than comparison groups.
Meyer et al.,	Australia / Victorian	Retrospective	To identify factors most strongly	n = 1,574; fallers n =	Fallers 81.6 ± 7.8	Male n = 846	T2DM-related mechanisms — Multiple	Falls; cognitive dysfunction; weight loss;	Falls were associated with cognitive dysfunction, recent

2020	at-home nursing care provider database	observational study	associated with falls among older people with diabetes receiving at-home care support services.	482, non-fallers n = 1,092	years; non-fallers 80.7 ± 7.6 years; eligibility ≥65 years	(53.7%); female n = 728 (46.3%)	diabetes-related mechanisms; gait impairment; cognitive impairment; sarcopenia / weight loss; urinary incontinence	gait issues; walking assistance; urinary incontinence; living alone; peripheral neuropathy; dizziness / syncope	weight loss, gait issues, need for walking assistance, urinary incontinence, and living alone. Lower-limb amputation appeared protective in this dataset.
Wang et al., 2021	Taiwan / outpatient clinics	Cross-sectional study	To explore risk factors for falls and gender-specific differences among older adults with diabetes attending outpatient clinics.	n = 485	65–80 years	NR	T2DM-related mechanisms — Balance dysfunction; muscle weakness; fear of falling	Falls; fall risk; handgrip strength; fear of falling; Timed Up and Go; physical capability	Female sex, weaker handgrip strength, and fear of falling were associated with falls overall. Fear of falling was especially important in males, while handgrip strength was important in females.
Mailliez et al., 2025	France / multidisciplinary fall consultation	Retrospective cohort study	To compare fall risk factors and geriatric syndromes between older fall-consultation patients with and without diabetes.	n = 1,520; diabetes subgroup n = 304	Overall 81.4 ± 6.4 years; diabetes group 79.4 ± 6.1 years	Female 72.2% overall	T2DM-related mechanisms — Multiple diabetes-related mechanisms; gait impairment; balance dysfunction; frailty; polypharmacy	Falls; recurrent falls; gait and balance disorders; frailty; cognitive function; polypharmacy; neuropathy; retinopathy; urinary disorders; functional dependency	Patients with diabetes had more geriatric syndromes and comorbidities than those without diabetes, including balance/gait disorders, cognitive impairment, frailty, functional dependency, and polypharmacy.
Varghese et al., 2024	United States / Health and Retirement Study	Longitudinal cohort study	To examine sex differences in the association of joint glycemic, blood pressure, and lipid control with two-year fall risk among older US adults with diabetes.	n = 2,829	Mean 72.2 years; ≥65 years	Female 54.7%; male 45.3%	T2DM-related mechanisms — Hypoglycemia / glycemic instability; HTN-related mechanisms — blood pressure control; Shared mechanisms — multimorbidity	Falls; glycemic control; systolic blood pressure; diastolic blood pressure; cholesterol control; polypharmacy; IADL	Joint cardiometabolic target control was not clearly associated with falls in women, but was associated with lower fall risk in men. Glycemic and blood-pressure control individually also appeared protective among men.
Sattar et al., 2017	Belgium	Case-control study	To determine whether transient hyperglycemia affected attentional functions and gait in patients with diabetes mellitus.	n = 36; hyperglycemia group n = 17, matched normoglycemic controls n = 19; gait subset n = 10	NR	NR	T2DM-related mechanisms — Hyperglycemia / glycemic instability; cognitive impairment; gait impairment	Transient hyperglycemia; attention tests; response time; tandem gait; center of pressure; total distance travelled	Transient hyperglycemia was associated with slower attentional responses and increased center-of-pressure travel during tandem gait, suggesting acute glycemic instability can impair attention and gait control.
Bayrak et al., 2025	Turkey / geriatric T2DM clinical sample	Cross-sectional study	To identify demographic, clinical, functional, and diabetes-related factors associated with fall risk in geriatric patients with T2DM.	n = 242; fallers n = 100 and non-fallers n = 142	Median age 70 years (IQR 66–74)	Male n = 123; female n = 119	T2DM-related mechanisms — neuropathy / sensory-feedback failure; hypoglycemia / glycemic instability; balance dysfunction; polypharmacy	Falls; fall risk; POMA; neuropathy; polypharmacy; insulin therapy; hypoglycemia; Mini Nutritional Assessment; HbA1c; glucose levels	Fall risk was associated with balance impairment, neuropathy, polypharmacy, insulin therapy, hypoglycemia, and poorer nutritional status. Glycemic markers were higher among fallers than non-fallers.
Hirata et al., 2018	Denmark	Experimental / biomechanical laboratory study	To examine postural control reorganization during sensory-challenging standing tasks in knee osteoarthritis patients with high versus low widespread pain sensitization.	n = 56	67.3 ± 8.1 years reported before exclusions; final analysis exact age unclear	Final analysis sex distribution NR	OA-related mechanisms — Pain; balance dysfunction; proprioceptive impairment	Pain sensitization; pressure pain threshold; postural sway; center of pressure; balance performance	High pain sensitization in knee osteoarthritis was linked to altered or posturally stiffer center-of-pressure behavior during challenging sensory conditions, suggesting sensory-motor reorganization relevant to balance control.
Zhao et al., 2025	China; first-author affiliation	Narrative review	To review effects of hypoglycemic agents on bone, muscle, and broader musculoskeletal health in type 2 diabetes.	N/A; comprehensive narrative review	N/A; review article	N/A; review article	T2DM-related mechanisms — Hypoglycemia / glycemic instability; medication-related musculoskeletal effects; sarcopenia / dynapenia	Medication exposure; muscle mass; muscle strength; sarcopenia; osteoporosis; fractures; falls; hypoglycemic agents	The review summarized evidence that hypoglycemic agents may influence musculoskeletal health through effects on bone density, fracture risk, muscle mass, and physical function, with implications for fall vulnerability in diabetes.
Mori et al., 2021	Japan / multicenter diabetes cohort	Cross-sectional study	To clarify the prevalence and clinical characteristics of sarcopenia and dynapenia in Japanese patients with type 1 and type 2 diabetes.	n = 1,328; T1DM n = 177, T2DM n = 645, controls n = 506	Participants aged ≥65 years reported in main older-adult analysis	NR	T2DM-related mechanisms — Sarcopenia / dynapenia; neuropathy / sensory-feedback failure; physical function decline	Falls; incidental falls; gait speed; grip strength; knee extension strength; skeletal muscle mass; sarcopenia; dynapenia; diabetic neuropathy; quality of life	Sarcopenia and dynapenia were more frequent in diabetes groups than controls among older participants. Sarcopenia or dynapenia was linked to lower physical quality of life and more incidental falls.
Çakici et al., 2023	Turkey	Cross-sectional study	To compare plantar fascia stiffness, fall risk, and gait speed between people with T2DM and healthy controls and examine stiffness-outcome associations.	n = 50 patients with T2DM; control group present but exact n unclear in extracted text	30–65 years eligibility	NR	T2DM-related mechanisms — Foot biomechanics / plantar pressure; gait impairment	Plantar fascia stiffness; fall risk; gait speed; 4-meter gait; Falls Efficacy Scale-International	People with T2DM had greater plantar fascia stiffness, worse fall-risk scores, and slower gait speed than controls. Higher plantar fascia stiffness was associated with higher fall risk and lower gait speed.
Downie et al., 2025	Australia	Experimental / biomechanical laboratory study	To investigate biomechanical differences in balance recovery after simulated forward falls in people with knee osteoarthritis versus asymptomatic controls under single- and dual-task conditions.	n = 63; knee OA n = 48 and controls n = 15	Knee OA 71.02 ± 6.76 years; controls 72.47 ± 4.81 years	Knee OA female 54%; controls female 27%	OA-related mechanisms — biomechanical compensation; balance dysfunction; gait alteration; cognitive dual-task challenge	Simulated falls; balance recovery; knee flexion; hip flexion; trunk flexion; dual-task balance; WOMAC; kinematics	People with knee OA used different balance-recovery kinematics after simulated forward falls, including greater knee flexion and reduced hip/trunk flexion. Dual-task challenges affected compensatory movement strategies.
Zhang et al., 2025	China / First Hospital of Qinhuangdao	Cross-sectional study	To identify risk factors for dysmobility syndrome in older adults with type 2 diabetes mellitus.	n = 262	Older adults; groups approximately 67.8–70.8 years	Male proportions by group: 50%, 45.2%, and 30%; exact overall sex distribution NR	T2DM-related mechanisms — Gait impairment; balance dysfunction; sarcopenia / dynapenia; falls	Falls; dysmobility syndrome; walking speed; muscle mass; muscle strength; osteoporosis; vitamin D; diastolic blood pressure	Dysmobility syndrome was common in older adults with T2DM. Older age, lower vitamin D, and history of falls were independent risk factors, and DMS groups showed more falls and poorer musculoskeletal indicators.
Hu et al., 2023	China	Cross-sectional observational study	To assess center-of-pressure shaking amplitude during static standing and analyze the effects of aging and diabetes on balance control.	n = 51; young adults n = 20, healthy older adults n = 16, diabetic patients n = 15	Young adults 27.65 ± 5.60 years; healthy older adults 58.88 ± 3.54 years; diabetic patients 58.33 ± 5.33 years	NR	T2DM-related mechanisms — Balance dysfunction; plantar center-of-pressure control	Static standing balance; center of pressure; plantar pressure; slope stance; confidence ellipse area; principal component analysis	Diabetic patients showed poorer static balance control than healthy young and older adults, with larger center-of-pressure ellipse area and increased minor-axis sway in several stance conditions.
Wang et al.,	China / Qilu Hospital	Retrospective	To identify risk factors and	n = 521; low muscle	≥60 years; normal	Normal muscle	T2DM-related mechanisms — Sarcopenia /	Low muscle mass; sarcopenia; diabetic	Older age, lower BMI, and diabetic sensorimotor

2026	of Shandong University	cross-sectional study	predictive performance for reduced muscle mass among older patients with type 2 diabetes.	mass n = 253, normal muscle mass n = 268	muscle mass 67.76 ± 5.56 years, low muscle mass 69.56 ± 6.33 years	mass: male 110/268; low muscle mass: male 157/253	dynapenia; diabetic sensorimotor polyneuropathy; medication-related muscle effects	sensorimotor polyneuropathy; BMI; age; insulin use; sulfonylurea use; DXA	polyneuropathy were independently associated with low muscle mass. Insulin use was linked to lower risk in men, whereas sulfonylurea use was linked to higher risk in women.
Handsaker et al., 2016	United Kingdom / Manchester	Experimental / biomechanical laboratory study	To examine the effects of diabetes and diabetic peripheral neuropathy on stepping accuracy during level-ground walking.	n = 36	Exact age NR in extracted text	NR	T2DM-related mechanisms — Neuropathy / sensory-feedback failure; gait impairment; visual-motor control	Stepping accuracy; foot-target contact; visual gaze behavior; gait; diabetic peripheral neuropathy; tripping risk	Participants with diabetes and those with diabetic peripheral neuropathy were less accurate when stepping onto targets than controls. Reduced motor control and altered gaze strategies may contribute to tripping risk.
Iliescu et al., 2025	Romania	Cross-sectional study	To evaluate whether diabetic neuropathy and its severity were associated with balance, mobility, and fear of falling in adults with T2DM.	n = 124	Older adults; exact age NR	NR	T2DM-related mechanisms — Neuropathy / sensory-feedback failure; balance dysfunction; fear of falling	Diabetic neuropathy; Berg Balance Scale; Timed Up and Go; Falls Efficacy Scale; fear of falling; SUDOSCAN; Michigan Neuropathy Screening Instrument	Diabetic neuropathy was associated with poorer balance and greater fear of falling. Neuropathy symptom scores predicted worse balance and mobility indicators.
D'Onofrio et al., 2026	Italy	Cross-sectional study	To examine whether diabetic neuropathy was associated with bone mineral density and fall risk in young-elderly adults with type 2 diabetes.	n = 144	Young-elderly adults; exact age NR in extracted text	NR	T2DM-related mechanisms — Neuropathy / sensory-feedback failure; fall risk; bone fragility-related mechanisms	Fall risk; diabetic neuropathy; bone mineral density; femoral neck BMD; total femur BMD; trabecular bone score	Participants with diabetic neuropathy had lower femoral-region bone mineral density and higher fall risk. The findings suggest neuropathy may link diabetes-related sensory impairment with bone fragility and fall vulnerability.
Lu et al., 2024	Taiwan / Tri-Service General Hospital	Cross-sectional study	To examine the association between osteoporosis and dynapenia in elderly patients with T2DM, with emphasis on female-specific risk.	n = 103 T2DM patients; male n = 43, female n = 60	Age range 50–80 years; median 68.0 years	Female n = 60; male n = 43	T2DM-related mechanisms — Sarcopenia / dynapenia; muscle weakness; bone fragility	Dynapenia; osteoporosis; handgrip strength; DXA; body composition; fall risk context	Osteoporosis and higher BMI were associated with dynapenia among female T2DM patients. The study indicates overlap between bone fragility and reduced muscle strength.
Nogueira et al., 2024	Brazil / Diabetes Care Center in Minas Gerais	Cross-sectional study	To evaluate sensory and motor aspects of balance in people with T2DM with and without peripheral diabetic neuropathy.	n = 51; T2DM with PDN n = 17, T2DM without PDN n = 17, controls n = 17	Adults >18 years; exact age NR	NR	T2DM-related mechanisms — Neuropathy / sensory-feedback failure; balance dysfunction; proprioceptive impairment	mCTSIB; Mini-BESTest; Timed Up and Go; TUG cognitive task; falls in last year; dizziness; peripheral diabetic neuropathy	Participants with peripheral diabetic neuropathy showed worse static and dynamic balance than diabetes-only and control groups. Diabetes without neuropathy also showed selected anticipatory and reactive balance deficits versus controls.
Tavares et al., 2024	Brazil / Basic Health Units, Serra Talhada, Pernambuco	Cross-sectional study	To compare balance, gait, functionality, and fall occurrence between adults and older adults with and without T2DM-associated peripheral neuropathy.	n = 70; T2DM with peripheral neuropathy n = 35, without T2DM n = 35	50–85 years	Both sexes; exact distribution NR in extracted text	T2DM-related mechanisms — Neuropathy / sensory-feedback failure; balance dysfunction; gait impairment	Falls in previous 6 months; Berg Balance Scale; Timed Up and Go; gait speed; functionality; plantar sensitivity; diabetic peripheral neuropathy	Participants with T2DM and peripheral neuropathy had worse balance, gait, and functionality indicators and a higher occurrence of falls than comparison participants without diabetes.
Khan et al., 2021	Denmark / Danish Center for Strategic Research in Type 2 Diabetes cohort and national registers	Cross-sectional study	To examine fall and fracture prevalence and their association with symptoms of diabetic polyneuropathy in recently diagnosed type 2 diabetes.	n = 5,359 questionnaire responders with neuropathy and fall data	Median age 65 years	NR	T2DM-related mechanisms — Neuropathy / sensory-feedback failure; fall-related fractures	Falls; recurrent falls; fractures; diabetic polyneuropathy symptoms; Michigan Neuropathy Screening Instrument	Possible diabetic polyneuropathy was strongly associated with falls, with higher prevalence ratios as fall number increased. Fracture association was slightly elevated but not statistically significant.
Kardm et al., 2025	Saudi Arabia / tertiary diabetes and neuromuscular rehabilitation setting	Cross-sectional study	To compare ankle proprioception and postural stability between individuals with T2DM and healthy controls and examine relationships with HbA1c and neuropathy.	n = 132; T2DM n = 66, controls n = 66	T2DM group 57.35 ± 6.24 years; controls 57.88 ± 6.47 years	Male n = 71; female n = 61	T2DM-related mechanisms — Neuropathy / sensory-feedback failure; proprioceptive impairment; balance dysfunction	Ankle proprioception; postural sway; center of pressure; postural stability; peripheral neuropathy; HbA1c; fall risk	Participants with T2DM had greater proprioceptive errors and postural instability than controls. Poor glycemic control and peripheral neuropathy were associated with worse proprioception and balance measures.
Özdemir et al., 2024	Turkey	Cross-sectional study	To investigate balance disorder and fall risk in patients with diabetic neuropathic pain and associated kinesiophobia and physical limitation.	n = 250; neuropathic pain n = 74, nociceptive/mechanical pain n = 96, no-pain control n = 90	Neuropathic pain 62.56 ± 9.81 years; nociceptive/mechanical pain 62.47 ± 11.67 years; control exact age NR	Female 52.3% in neuropathic pain group; full distribution NR	T2DM-related mechanisms — Neuropathy / sensory-feedback failure; pain burden; kinesiophobia / fear of movement; balance dysfunction	Neuropathic pain; kinesiophobia; physical activity; Berg Balance Scale; Falls Efficacy Scale-International; fall risk	Patients with diabetic neuropathic pain showed poorer balance, lower physical activity, higher kinesiophobia, and increased fall risk versus comparison groups. Fall risk rose with balance impairment.
Lopatin et al., 2025	United States; first-author affiliation	Narrative review	To synthesize how type 2 diabetes affects sensorimotor control of balance through peripheral neuropathy, retinopathy, and vestibular dysfunction.	N/A; narrative review	N/A; review article	N/A; review article	T2DM-related mechanisms — Neuropathy / sensory-feedback failure; visual impairment; vestibular dysfunction; balance dysfunction	Balance control; diabetic peripheral neuropathy; diabetic retinopathy; vestibular dysfunction; proprioception; falls; fear of falling	The review described how T2DM-related peripheral neuropathy, retinopathy, and vestibular dysfunction can disrupt the sensory inputs required for balance, increasing postural instability and fall vulnerability.
Riandini et al., 2020	Singapore	Cross-sectional study	To examine associations between balance confidence and fall risk among patients with diabetic peripheral neuropathy.	n = 146 analysed with DPN; parent cohort n = 236	NR	NR	T2DM-related mechanisms — Neuropathy / sensory-feedback failure; fear of falling; balance dysfunction	Fall risk; balance confidence; Activities-specific Balance Confidence Scale; functional reach; body sway velocity; toe extensor strength; diabetes duration; BMI	Twenty percent of DPN patients were at increased fall risk. Lower balance confidence was associated with higher odds of fall risk, and psychological confidence appeared more important than objective functional performance.
Khan et al., 2021	Denmark	Cross-sectional study	To estimate falls in people with type 2 diabetes and examine motor dysfunction, postural instability, and diabetic polyneuropathy in	n = 131; T2DM with DPN n = 54, T2DM without DPN n = 38, controls n = 39	18–80 years	T2DM participants female n = 36; controls female n	T2DM-related mechanisms — Neuropathy / sensory-feedback failure; balance dysfunction; gait impairment	Falls; diabetic polyneuropathy; postural instability; motor function; walking endurance; sit-to-stand; balance performance	Individuals with type 2 diabetes reported more falls than controls. Fallers showed poorer walking endurance, sit-to-stand performance, and balance-related measures, while DPN alone did not fully explain fall status.

			relation to fall risk.			= 19			
La Scaleia et al., 2024	Italy / Sapienza University and IRCCS Santa Lucia Foundation, Rome	Experimental / biomechanical laboratory study	To assess whether impaired vestibular perception of self-motion was associated with postural instability and falls in older adults with type 2 diabetes.	n = 113; fall follow-up data n = 95	65–75 years; mean 68.8 ± 3.3 years	Female n = 28; male n = 85	T2DM-related mechanisms — Vestibular dysfunction; balance dysfunction; multiple diabetes-related mechanisms	Falls; vestibular motion thresholds; Modified Romberg Test; Berg Balance Scale; postural sway; glycemic control; hypertension	Participants with poorer vestibular thresholds had greater postural instability and higher risk of reported falls during follow-up. Longer diabetes duration, poorer glycemic control, and hypertension were associated with poorer vestibular thresholds.
Rasmussen & Dal, 2019	Denmark; first-author affiliation	Literature review / narrative review based on systematic search	To review diabetes-related risk factors for falls and fractures, including bone fragility, body composition, postural control, gait deficits, neuropathy, cardiovascular disease, and comorbidities.	53 studies included after full-text eligibility screening	People with diabetes; exact age NR	Participant-level sex/gender NR	T2DM-related mechanisms — multiple diabetes-related mechanisms; gait impairment; balance dysfunction; sarcopenia / dynapenia; neuropathy	Falls; fractures; bone fragility; sarcopenia; body composition; gait deficits; neuropathy; postural control; polypharmacy; orthostatic hypotension	The review summarized that fall risk in diabetes is multifactorial and includes gait/balance impairment, neuropathy, sarcopenia, comorbidities, orthostatic symptoms, and medication-related factors beyond bone fragility alone.
Komalasari et al., 2024	Indonesia / clinical T2DM sample	Cross-sectional study	To compare balance performance, falls efficacy, social participation, and quality of life between people with T2DM with and without vestibular dysfunction.	n = 322; T2DM with VD n = 161 and T2DM without VD n = 161	Adults with T2DM; exact age NR in extracted text	Sex/gender NR in extracted text	T2DM-related mechanisms — vestibular dysfunction; balance dysfunction; falls efficacy; social participation limitation	Vestibular dysfunction; balance performance; mCTSIB; Timed Up and Go; Functional Gait Assessment; falls efficacy; social participation; quality of life	T2DM participants with vestibular dysfunction had poorer balance performance, lower falls efficacy, greater social participation restriction, and lower quality of life than those without vestibular dysfunction.
Piker & Romero, 2019	United States; first-author affiliation	Narrative review	To review evidence on diabetes-related vestibular system involvement and its relevance to dizziness, balance, and fall-related outcomes.	N/A; narrative review	N/A; narrative review	N/A; narrative review	T2DM-related mechanisms — Vestibular dysfunction; balance dysfunction; multiple diabetes-related mechanisms	Diabetes mellitus; vestibular function; dizziness; balance; falls; vestibular testing	The review summarized evidence that diabetes may impair vestibular function and contribute to dizziness, imbalance, and fall vulnerability, but emphasized that available evidence remains incomplete and heterogeneous.
Knox et al., 2021	United States	Prospective cohort study / secondary analysis	To determine whether hip OA signs and symptoms were associated with fall risk over 1 year among older adults with chronic low back pain.	n = 210	Non-multiple fallers 69.85 ± 6.74 years; multiple fallers 68.97 ± 7.38 years	Female n = 106 (50.5%) in non-multiple fallers; female n = 22 (55.0%) in multiple fallers	OA-related mechanisms — Hip OA signs/symptoms; pain; balance confidence; physical function decline	Falls; multiple falls; hip pain; hip internal rotation; hip OA impairment burden; balance confidence; low back pain disability	Higher hip OA impairment burden was associated with increased fall risk and higher odds of multiple falls among older adults with chronic low back pain.
Sonobe et al., 2024	Japan / Locomotive Syndrome and Health Outcomes in Aizu Cohort Study (LOHAS)	Cross-sectional study	To identify factors associated with fall history and determine whether radiographic knee OA severity contributed independently to falls.	n = 1,083 analytical sample	≥60 years; exact mean age NR in extracted text	NR in extracted text	OA-related mechanisms — Pain; physical function decline; mobility limitation; depression	Fall history; radiographic knee OA severity; knee pain; knee crepitus; TUG; one-leg standing test; depression	Knee pain, decreased mobility, and depression were associated with fall history. Radiographic knee OA severity was not independently associated with fall risk.
Chan et al., 2024	Hong Kong / laboratory-based stair-walking assessment	Experimental / biomechanical laboratory study	To compare whole-body angular momentum between people with and without knee osteoarthritis during stair ascent and descent.	n = 15; knee OA n = 7, controls n = 8	Knee OA 56.3 ± 6.8 years; controls 57.9 ± 6.7 years	Knee OA male/female 2/5; controls male/female 4/4	OA-related mechanisms — Biomechanical compensation; dynamic balance during stair negotiation	Whole-body angular momentum; stair ascent; stair descent; knee kinetics; hip flexion; foot progression angle; dynamic balance	Participants with knee OA showed greater sagittal-plane angular momentum during stair ascent and descent and greater transverse-plane angular momentum during descent, indicating altered dynamic balance strategies.
Ogawa et al., 2020	United States / MOBILIZE Boston Study II	Cross-sectional study	To investigate whether chronic musculoskeletal pain distribution was associated with gait impairment through an attention-mediated pathway in community-living older adults.	n = 302 completed gait assessment; multivariable models n = 292	Mean 84 years; range 71–101 years	Female n = 194 (64.24%); male n = 108 (35.76%)	Shared / multimorbidity-related mechanisms — Pain burden; cognitive impairment; physical function decline	Pain distribution; gait speed; stride length; double support; swing time; gait variability; dual-task gait; selective attention	Pain distribution was associated with slower gait speed and poorer gait characteristics across single- and dual-task walking. Selective attention partly mediated several pain-gait associations, but dual-task costs did not differ clearly by pain group.
Hughes-Olivera et al., 2018	United States	Experimental / biomechanical laboratory study	To determine associations of limb and gender with temporal gait parameters and gait variability in end-stage unilateral ankle osteoarthritis.	n = 242 end-stage unilateral ankle OA patients	NR	Female n = 132; male n = 110	OA-related mechanisms — Gait alteration; biomechanical compensation	Temporal gait parameters; gait variability; stride time; swing time; stance time; double support time	Women had longer stride times and higher stride and stance-time variability than men, suggesting potentially greater gait-instability-related fall risk. Both sexes showed antalgic adaptations by reducing loading on the affected limb.
Bezalel et al., 2021	Israel	Case-control study	To examine whether unilateral knee osteoarthritis affected central and peripheral components of voluntary step execution compared with age-matched controls.	n = 43; knee OA n = 21, controls n = 22	≥65 years	NR	OA-related mechanisms — Biomechanical compensation; movement limitation; cognitive impairment	Voluntary step execution; initiation phase; preparatory phase; swing phase; dual-task stepping; fall risk	Knee OA participants moved more slowly than controls during preparatory and swing phases, especially in forward stepping. Dual-tasking slowed pre-movement initiation across groups, indicating a fall-relevant rapid-stepping deficit.
Manlapaz et al., 2019	New Zealand; first-author affiliation	Systematic review	To examine evidence on risk factors for falls in adults with knee osteoarthritis.	11 studies; total participants NR	Adults with knee OA; older adults included; exact age NR	Participant-level sex/gender NR	OA-related mechanisms — balance dysfunction; muscle weakness; pain; proprioceptive impairment; multiple OA-related mechanisms	Falls; balance performance; muscle strength; knee pain; symptomatic joints; proprioception; walking aid use; comorbidities	The review identified impaired balance, reduced knee muscle strength, symptomatic joints, and comorbidities as fall-related factors in knee OA. Evidence for pain, instability, proprioception, and walking aids was limited or conflicting.
Ma et al., 2025	China / laboratory-based gait analysis	Experimental / biomechanical laboratory study	To compare intrasubject gait variability during stair walking between individuals with knee osteoarthritis and healthy controls.	n = 169; knee OA n = 116 and controls n = 53	Knee OA group 58.49 ± 5.97 years; control group 59.00 ± 5.97 years	Knee OA: female 102, male 14; controls: female 32, male 21	OA-related mechanisms — Gait alteration; biomechanical compensation; balance-related stair-walking variability	Stair walking; gait variability; kinematics; kinetics; joint angles; joint moments; joint power	Participants with knee osteoarthritis showed greater kinematic variability and selected kinetic variability during stair walking than controls, suggesting altered motor control and biomechanical adaptation during a fall-relevant task.
Iijima et al., 2020	Japan / community-dwelling	Cross-sectional study	To examine whether low back pain was associated with recurrent falls	n = 189	Age range 61–90 years	Female 148/189 (78.3%); male	OA-related mechanisms — Pain burden; recurrent falls; physical function decline;	Low back pain; recurrent falls; knee osteoarthritis; pain intensity; physical	Low back pain, especially moderate-to-severe low back pain, was associated with recurrent falls among people with

	adults with knee osteoarthritis		among people with knee osteoarthritis.			41/189 (21.7%)	multiple OA-related mechanisms	function; gait speed; balance-related outcomes	knee osteoarthritis. The finding supports pain burden as a relevant fall-related mechanism.
Lo-Ciganic et al., 2017	United States / Osteoarthritis Initiative	Longitudinal cohort study	To assess whether different analgesic categories were associated with subsequent recurrent falls among participants with or at risk of knee osteoarthritis.	n = 4,231	Age range 45–79 years	Sex/gender NR in extracted text	OA-related mechanisms — pain; medication exposure; recurrent falls; opioid / antidepressant exposure	Recurrent falls; analgesic use; opioids; antidepressants; NSAIDs; OTC pain medication; knee OA; pain severity; depression severity	Participants using opioids or antidepressants had increased risk of recurrent falls in the following year after adjustment. Findings support medication-related fall pathways among people with or at risk of knee OA.
Alenazi et al., 2023	United States / Healthcare Enterprise Repository for Ontological Narration (HERON) electronic medical records	Retrospective observational study	To examine whether generalized and localized osteoarthritis were associated with falls and whether chronic diseases and medications contributed to fall risk.	n = 760	≥65 years; exact mean age NR in extracted text	Female approximately 490/760; exact sex distribution should be manually confirmed from table	OA-related mechanisms — Multiple OA-related mechanisms; pain; medication exposure; comorbidity-related fall risk	Falls; recurrent falls; generalized osteoarthritis; localized osteoarthritis; hypertension; diabetes; neuropathy; depression; sleep disorders; medication exposure	Generalized osteoarthritis was associated with greater odds of falls and recurrent falls. Hypertension, neuropathy, sleep disorders, and selected medications also contributed to fall-related risk patterns.
Alenazi, 2026	United States / Osteoarthritis Initiative	Secondary analysis of cohort data	To examine how knee osteoarthritis and multimorbidity interact in relation to longitudinal fall risk.	n = 4,449	Age range 45–79 years	Sex reported in source dataset; exact distribution NR in extracted text	OA-related mechanisms — Multimorbidity; pain; physical function decline; multiple OA-related mechanisms	Falls; knee osteoarthritis; multimorbidity; chronic conditions; pain; physical function	Having one chronic disease with or without knee osteoarthritis was associated with increased fall risk. Multimorbidity in people with knee osteoarthritis was associated with a higher number of falls over follow-up.
Rosadi et al., 2022	Indonesia / Malang	Cross-sectional study	To identify significant predictors of falls in patients with knee osteoarthritis.	n = 372	≥50 years	NR	OA-related mechanisms — Proprioceptive impairment; pain; physical function decline; diabetes-related comorbidity	Falls; knee proprioception; knee range of motion; KOOS symptoms; fear of falling; diabetes mellitus; BMI; TUG; chair stand test	Better knee proprioception and range of motion were protective against falls, whereas higher KOOS symptoms, fear of falling, diabetes, BMI, and low back pain were associated with fall risk in knee OA.
Huang et al., 2024	China	Experimental / biomechanical laboratory study	To determine whether ankle inversion proprioceptive acuity during walking was impaired in people with knee osteoarthritis and whether sex differences existed.	n = 66; knee OA n = 32, healthy controls n = 34	KOA median 52.5 years; controls median 49.0 years	KOA female/male n = 17/15; controls female/male n = 17/17	OA-related mechanisms — Proprioceptive impairment; gait alteration; biomechanical compensation	Ankle inversion movement discrimination; walking proprioception; knee OA; KOOS; fall risk proxy	People with knee OA showed lower ankle inversion movement discrimination sensitivity during walking than controls. The impairment was similar in men and women and may represent a proprioceptive pathway relevant to fall risk.
Wilfong et al., 2023	Canada / Canadian Longitudinal Study on Aging	Prospective cohort study	To examine whether knee osteoarthritis was associated with increased fall risk in a population-based longitudinal cohort.	n = 21,710	Age range 45–85 years	NR in extracted text	OA-related mechanisms — Pain; physical function decline; balance dysfunction; injurious falls	Falls; injurious falls; recurrent injurious falls; knee osteoarthritis; urinary incontinence; balance; depression; physical function; pain	Knee osteoarthritis independently contributed to increased fall risk. Previous falls, previous fractures, urinary incontinence, balance limitation, depression, and pain were also important predictors of future falls.
Freitas et al., 2023	Brazil / Centro Universitário de Anápolis	Experimental / biomechanical laboratory study	To analyze postural control in patients with unilateral knee osteoarthrosis and examine its relationship with fall risk.	n = 40; knee osteoarthrosis group n = 20 and control group n = 20	OA group 71.59 ± 14.09 years; control group 71.09 ± 13.08 years	Table reports sex by group; exact male/female values require manual confirmation	OA-related mechanisms — Balance dysfunction; proprioceptive impairment; postural control; fall risk	Postural control; center of pressure displacement; balance performance; fall risk; knee osteoarthrosis	The knee osteoarthrosis group showed greater center-of-pressure displacement than controls, particularly with eyes open, indicating poorer postural control that may be relevant to fall risk.
Ikutomo et al., 2018	Japan / Masuhara Clinic and community controls	Cross-sectional study	To determine fall incidence in women with end-stage hip osteoarthritis and identify physical factors associated with falling.	n = 265; hip OA n = 153, healthy controls n = 112	Hip OA 64.0 ± 9.1 years; controls 64.1 ± 9.3 years	Female 100%	OA-related mechanisms — Gait alteration; muscle weakness; biomechanical compensation; physical function decline	Falls; fall-related injuries; fracture; limping; knee extensor strength; hip abductor strength; 10-m walking time; Harris Hip Score	Women with end-stage hip OA had higher fall incidence than controls. In the hip OA group, limping and lower knee extensor strength were associated with falls.
Mat et al., 2019	Malaysia / Malaysian Elders Longitudinal Research (MELoR) Study	Cross-sectional study	To evaluate the relationship between knee pain severity and falls and examine functional and psychological mediators among older Malaysian adults.	n = 1,212	≥55 years	Female n = 688; male n = 524	OA-related mechanisms — Pain; physical function decline; depression / psychological symptoms; balance dysfunction	Falls; knee pain; knee pain severity; Timed Up and Go; Katz ADL; Lawton IADL; depression; anxiety; stress	Knee pain was associated with higher fall risk. Mediation analyses suggested that functional impairment and psychological symptoms, especially depression and stress, partly explained the knee pain–falls relationship.
Grønne et al., 2024	Denmark / Good Life with Osteoarthritis in Denmark (GLA:D) registry	Cross-sectional study	To estimate the prevalence of concerns about falling in people with knee or hip osteoarthritis and examine associations with pain, function, and psychological factors.	n = 7,442	Mean age 67 years (SD 9.6)	Female 67%; male 33%	OA-related mechanisms — Kinesiophobia / fear of movement; pain; physical function decline; psychological symptoms	Concerns about falling; pain intensity; physical function; chair-stand performance; fear of movement; EQ-5D; knee/hip osteoarthritis	Concerns about falling were common in people with knee or hip osteoarthritis. Higher pain, poorer physical function, and psychological factors such as fear of movement were associated with greater concern.
Endo et al., 2024	Japan / single rehabilitation facility	Pilot cross-sectional study	To provide preliminary evidence on factors associated with stiff knee gait among patients with knee osteoarthritis undergoing rehabilitation.	n = 21; SKG+ n = 9, SKG− n = 12	NR in current PDF page	NR in current PDF page	OA-related mechanisms — Gait alteration; pain; muscle weakness; stiffness / movement limitation	Stiff knee gait; knee flexion excursion; pain intensity; quadriceps strength; knee range of motion; WOMAC; gait speed; FES-I	Stiff knee gait was associated with greater pain intensity, reduced quadriceps strength, and reduced knee flexion range of motion. The current PDF appears to contain only the first page/abstract.
Güngör Demir et al., 2021	Turkey / Physical Therapy and Rehabilitation outpatient settings	Cross-sectional study	To investigate neuropathic pain in knee OA and its associations with clinical, anthropometric, physical function, quality-of-life, and	n = 200; neuropathic pain group n = 98, non-neuropathic pain group n = 102	Neuropathic pain group 61 (50–70) years; non-neuropathic pain group 57.5 (50–70)	Neuropathic pain group female n = 81/98; non-neuropathic	OA-related mechanisms — Neuropathic/nociplastic pain; balance dysfunction; physical function decline	Neuropathic pain; knee OA; posturography; fall risk; stability indices; WOMAC; physical function; quality of life	Nearly half of knee OA patients had neuropathic pain. Neuropathic pain was associated with worse clinical severity, lower physical function and quality of life, and impaired stability indices, although fall risk was similar

			posturography outcomes.		years	pain group female n = 76/102			between groups.
Alenazi, 2025	United States / Osteoarthritis Initiative	Prospective longitudinal cohort study / secondary analysis of Osteoarthritis Initiative data	To examine whether baseline multisite pain was associated with longitudinal fall risk and recurrent falls among older adults with or at risk of knee osteoarthritis.	n = 2,585	Older adults with or at risk of knee OA; exact mean age NR in extracted text	NR	OA-related mechanisms — Pain burden; multisite pain; recurrent falls; physical function decline	Falls; recurrent falls; fall count; multisite pain; knee osteoarthritis; PASE; depressive symptoms; comorbidities	Baseline pain at two sites and at three or more sites was associated with increased fall incidence and fall number. Pain at three or more sites was associated with recurrent falls.
Ko et al., 2018	United States / Baltimore Longitudinal Study of Aging	Cross-sectional analysis of cohort data	To examine usual-speed gait parameters by fall history and knee pain status in older adults.	n = 439	Age range 60–92 years	Female n = 211; male n = 228	OA-related mechanisms — Gait alteration; pain; biomechanical compensation	Falls; fall history; knee pain; gait speed; stride width; double support time; hip range of motion; WOMAC-derived knee pain	Fallers had wider stride width and longer double support time than nonfallers. Knee pain modified fall-related gait patterns, indicating altered gait compensation in painful knees.
Harris et al., 2023	United States / Osteoarthritis Initiative	Secondary analysis of cohort data / longitudinal cohort study	To examine whether radiographic knee osteoarthritis severity was associated with recurrent falls in middle-aged and older adults enrolled in the Osteoarthritis Initiative.	n = 3,972	Mean age 63 years; age range 45–79 years	Female 58%; male 42%	OA-related mechanisms — Balance dysfunction; physical function decline; recurrent falls	Recurrent falls; radiographic knee OA severity; Kellgren–Lawrence grade; knee pain; knee confidence; knee range of motion; medication exposure	Older adults aged ≥65 years with possible or definite radiographic knee OA had higher odds of recurrent falls than peers without radiographic OA. Associations varied by OA severity and age stratum.
Mat et al., 2017	Malaysia	Case-control study	To examine whether fear of falling and psychological symptoms mediated the relationship between osteoarthritis severity and falls in older adults.	n = 389; fallers n = 229, non-fallers n = 160	Mean 72.2 ± 6.1 years	Female proportion NR in overall sample; OA subgroup table reports sex by faller status	OA-related mechanisms — Pain; kinesiophobia / fear of movement; psychological symptoms; physical function decline	Falls; injurious falls; WOMAC; fear of falling; DASS-21 depression/anxiety/stress; osteoarthritis severity	Fear of falling was associated with falls in older adults with radiological or clinical OA. Psychological symptoms, especially depressive symptoms, partly explained the relationship between OA symptom severity and fall risk.
Levinger et al., 2016	Australia	Experimental / biomechanical laboratory study	To compare trunk and lower-limb biomechanical responses during forward induced falls under normal and dual-task conditions in people with and without knee osteoarthritis.	n = 39; knee OA n = 24, control n = 15	Mean 72.4 ± 4.8 years	Knee OA: female n = 12 (50%); control: female n = 4 (26.7%); total female n = 16 (41.0%), male n = 23 (59.0%)	OA-related mechanisms — Balance dysfunction; biomechanical compensation; cognitive impairment	Induced falls; balance recovery; center of mass velocity; margin of stability; step velocity; hip/knee/ankle joint angles; dual-task balance	Participants with knee OA had reduced center-of-mass velocity, lower margin of stability, slower step velocity, and reduced hip/knee motion during induced falls. Dual-task conditions further impaired balance recovery responses.
Pater et al., 2016	United States	Experimental / biomechanical laboratory study	To compare recovery-step kinematics after large forward-directed perturbations in women with and without self-reported knee osteoarthritis.	n = 50; knee OA n = 25, control n = 25	Knee OA 60.8 ± 6.9 years; controls 60.4 ± 7.8 years	Female 100%	OA-related mechanisms — Biomechanical compensation; balance dysfunction; gait alteration	Induced falls; recovery step length; trunk flexion angle; trunk angular velocity; knee pain; KOOS	Knee OA was associated with impaired recovery-step kinematics after laboratory-induced trip perturbation, especially shorter recovery steps and altered trunk flexion velocity. Fall proportions did not differ significantly between OA and controls.
Ekediegwu et al., 2022	Nigeria / outpatient orthopaedic clinics in Anambra State	Cross-sectional study	To examine demographic and disease characteristics associated with pain intensity, kinesiophobia, balance, and fall self-efficacy among people with knee or hip OA.	n = 70	Mean age 59.91 ± 11.12 years	Female n = 59 (84.3%); male n = 11 (15.7%)	OA-related mechanisms — Pain; kinesiophobia / fear of movement; balance dysfunction; fall self-efficacy	Pain intensity; kinesiophobia; fall self-efficacy; Timed Up and Go; balance performance; comorbidity; OA site	Pain intensity, kinesiophobia, fall self-efficacy, and balance were interrelated in people with OA. Comorbidity and adaptive behaviours helped predict kinesiophobia and fall-related proxy outcomes.
Shen et al., 2024	China	Cross-sectional study	To examine how proprioception, plantar tactile sensation, pain, joint range of motion, and strength correlated with balance control among older adults with and without knee OA.	n = 240; knee OA n = 124, controls n = 116	Knee OA group 68.8 ± 4.0 years; control group 67.9 ± 3.5 years	Knee OA female n = 84, male n = 40; controls female n = 72, male n = 44	OA-related mechanisms — Proprioceptive impairment; pain; plantar tactile sensation; muscle weakness; balance dysfunction	Berg Balance Scale; proprioception; plantar tactile sensation; pain; ROM; strength; knee OA	Older adults with knee OA had lower BBS scores, worse proprioception and tactile thresholds, smaller ROM, and lower strength. Proprioception showed the strongest relationship with balance control.
Alkan Melikoğlu & Kul, 2018	Turkey	Cross-sectional study	To determine fall risk in patients with knee osteoarthritis using computerized balance assessment and evaluate related fall-risk factors.	n = 130; knee OA n = 100, comparison group n = 30	Age range 40–75 years	Sex/gender NR in extracted text	OA-related mechanisms — balance dysfunction; pain; physical function decline	Fall risk; balance performance; pain; WOMAC; Falls Efficacy Scale-International; knee OA grade; physical function	Knee OA patients showed higher fall-risk measures than controls. Fall risk was related to pain, functional impairment, and balance-related factors, with worse findings in more advanced knee OA grades.
Manckoundia et al., 2025	France; first-author affiliation	Literature review	To review causes, assessment, and management of balance and gait disorders in older adults.	N/A; literature review	Older adults; exact age NR	N/A; literature review	Shared / multimorbidity-related mechanisms — Multiple shared mechanisms; vestibular dysfunction; medication exposure; orthostatic hypotension; musculoskeletal disease	Falls; fall risk; gait disorders; balance disorders; orthostatic hypotension; vestibular function; medication exposure; sensory impairment	The review summarized multiple contributors to balance and gait disorders in older adults, including diabetes, vestibular disorders, orthostatic hypotension, osteoarticular disease, neurological disease, and medication-related mechanisms.
Kamitani et al., 2019	Japan / Locomotive Syndrome and Health Outcomes in Aizu Cohort Study (LOHAS)	Prospective cohort study	To examine whether discrepancy between self-reported and performance-based physical functioning predicted future falls in community-dwelling older adults.	n = 1,379	≥65 years	NR	Shared / multimorbidity-related mechanisms — Physical function decline; ADL decline	Falls; Timed Up and Go; SF-12 physical functioning; self-reported physical function; performance-based physical function	During follow-up, 22% reported a fall. Participants with low self-reported physical functioning had higher fall risk, while performance-based functioning alone was not consistently associated after accounting for self-perception.

Kammerlind et al., 2016	Sweden / community-dwelling older adults	Cross-sectional study	To determine the prevalence of mild and substantial dizziness and associated health, function, medication, and fall-related factors in community-dwelling older adults.	n = 305	Age range 75–90 years; mean age 81 years	Sex/gender NR in extracted text	HTN-related mechanisms — dizziness / syncope; medication burden; balance dysfunction; diabetes-related dizziness context	Dizziness; falls; fear of falling; blood pressure; diseases; medication burden; antihypertensive drugs; physical activity; quality of life; physical performance	Substantial dizziness was associated with more falls, fear of falling, diabetes, stroke/TIA, heart disease, greater drug burden, lower physical performance, and poorer quality of life.
Zhang et al., 2021	China / Beijing Tongren Hospital	Cross-sectional study	To examine the relationship between chronic conditions and balance disorders in outpatients with dizziness.	n = 332	Mean age 52.92 ± 14.07 years	Male 121 (36.4%); female 211 (63.6%)	HTN-related mechanisms — Dizziness / syncope; balance dysfunction; chronic disease burden; diabetes and hypertension as comorbid mechanisms	Dizziness; balance disorders; computerized dynamic posturography; hypertension; diabetes mellitus; chronic conditions	Chronic conditions were more common among patients with abnormal balance. Hypertension and diabetes were significantly associated with abnormal balance in descriptive analyses, especially among older participants.
Borges et al., 2020	Brazil / Brazilian Longitudinal Study of Aging (ELSI-Brazil) baseline	Cross-sectional study	To estimate dynapenia prevalence and identify factors associated with dynapenia in Brazilian older adults.	n = 8,396	Mean age 62.1 years	Female 53.3%; male 46.7%	Shared / multimorbidity-related mechanisms — Sarcopenia / dynapenia; physical function decline; ADL decline; falls	Dynapenia; handgrip strength; gait speed; falls; ADL; chronic diseases; physical activity	Dynapenia prevalence was 17.2% overall and higher in older age groups. Dynapenia was associated with lower gait speed, ADL limitation, falls, chronic disease burden, and lower physical activity.
Afrin et al., 2016	Finland / Kuopio Osteoporosis Risk Factor and Prevention Study (OSTPRE)	Prospective cohort study	To examine whether multimorbidity predicted different types of falls in postmenopausal women.	n = 10,594	47–56 years at baseline	Female 100%	Shared / multimorbidity-related mechanisms — Multimorbidity	Falls; indoor falls; outdoor falls; injurious falls; medication exposure; chronic conditions	Greater number of chronic conditions was associated with higher fall risk, with stronger associations for injurious and indoor falls. Multimorbidity predicted falls differentially by fall type.
Hamacher et al., 2019	Germany / community-recruited older adults	Cross-sectional study	To examine dynamic gait stability and identify physical, sensory, cognitive, pain, and health-related factors influencing gait stability in older adults.	n = 102	Mean age 72 ± 4.5 years; eligibility ≥65 years	Female n = 52; male n = 50	Shared / multimorbidity-related mechanisms — Physical function decline; gait impairment; pain burden; fear of falling	Gait stability; gait variability; dual-task walking; balance performance; pain; fear of falling; depression; osteoarthritis; diabetes; hypertension	Gait stability and gait variability were associated with multiple functional and sensorimotor factors. The study supports gait variability and stability as fall-risk-related proxy outcomes in older adults.
Gadelha et al., 2018	Brazil	Prospective cohort study	To assess whether sarcopenia stages were associated with incident falls over 18 months in older community-dwelling women.	n = 246 baseline assessed; n = 195 followed over 18 months	Baseline 68.1 ± 6.2 years; followed sample 68.2 ± 6.1 years; eligibility 60–85 years	Female 100%	Shared / multimorbidity-related mechanisms — Sarcopenia / dynapenia; physical function decline	Incident falls; multiple falls; sarcopenia stage; muscle mass; muscle strength; Timed Up and Go; lower-limb pain; polypharmacy	Severe sarcopenia was independently associated with higher fall incidence over about 18 months. Fall proportions increased substantially across worsening sarcopenia stages.
Hicks et al., 2020	Australia / Sydney Memory and Aging Study	Prospective cohort study	To examine physical, psychological, and medical mediators of the relationship between knee pain and fall risk in older community-dwelling adults.	n = 333	Mean age 83.3 ± 4.27 years in knee pain group; 83.2 ± 4.06 years in no-pain group; eligibility ≥70 years	Female n = 176 (52.9%); male n = 157 (47.1%)	OA-related mechanisms — Pain; muscle weakness; balance dysfunction; fear of falling	Multiple falls; knee pain; knee extension torque; postural sway; Falls Efficacy Scale-International; TUG; gait speed; medications	Knee pain doubled the risk of multiple falls over 12 months. Concern about falling, knee extension torque, and postural sway mediated part of the knee pain-fall association.
Roitto et al., 2023	Finland / Helsinki long-term care facilities	Longitudinal cohort study	To explore how medication use was associated with falls, fall-related consequences, and all-cause mortality among long-term care residents.	n = 532	≥65 years	NR	Shared / multimorbidity-related mechanisms — Polypharmacy / fall-risk-increasing drugs	Falls; fall injuries; fractures; hospitalizations; polypharmacy; excessive polypharmacy; opioids; anticholinergic medications; mortality	Fall rates increased with medication burden. Excessive polypharmacy, opioids, and anticholinergic medications predicted falls, and use of >10 medications predicted all-cause mortality.
Maly et al., 2019	Czech Republic / four hospitals in South Bohemia	Prospective observational study	To analyze the effect of fall-risk-increasing drugs and other drug-related factors on inpatient falls through clinical pharmacy service.	n = 280 inpatient falls	Mean age 77.0 years	NR	Shared / multimorbidity-related mechanisms — Polypharmacy / fall-risk-increasing drugs; medication-related adverse effects	Inpatient falls; FRIDs; total medications; drug-related fall causes; clinical pharmacist assessment; physician assessment	Among 280 inpatient falls, patients had high medication burden and frequent FRID exposure. Clinical pharmacists more often judged pharmacotherapy as fall-related than physicians.
Murtezani et al., 2025	Kosovo / nursing homes in Pristina	Cross-sectional study	To investigate medication use and its association with falls among older adults.	n = 125	Mean age 76.52 ± 7.83 years	Female 66%; male 34%	Shared / multimorbidity-related mechanisms — Polypharmacy / fall-risk-increasing drugs; medication exposure	Falls; medication exposure; polypharmacy; fall-risk-increasing drugs; comorbidities	Falls were reported by 26% of participants. Fallers had greater exposure to medication burden and fall-risk-related drug classes, supporting medication exposure as a relevant fall-related mechanism.
Salkın et al., 2025	Turkey / Fırat University Hospital	Cross-sectional study	To examine balance, kinesiophobia, and mobility in older adults exposed to polypharmacy.	n = 75	Age groups: 65–70 years n = 45; 71–75 n = 15; 76–80 n = 2; ≥81 n = 13	Female 39 (52.0%); male 36 (48.0%)	Shared / multimorbidity-related mechanisms — Polypharmacy / fall-risk-increasing drugs; kinesiophobia / fear of movement; balance dysfunction; mobility limitation	Fall history; Berg Balance Scale; Tampa Kinesiophobia Scale; Timed Up and Go; polypharmacy; assistive device use; chronic disease	Participants with fall history had lower balance scores and higher kinesiophobia. Chronic disease and medication burden were associated with poorer balance, greater kinesiophobia, and slower mobility.
Sengul Aycicek et al., 2021	Turkey / Gazi University Faculty of Medicine, geriatric outpatient clinic	Cross-sectional study	To evaluate the effects of polypharmacy and inappropriate drug use on balance and fall risk in older adults.	n = 101; polypharmacy group n = 51 and control group n = 50	Median 72 years; range 65–86 years	Female 56/101 (55.4%); male 45/101 (44.6%)	Shared / multimorbidity-related mechanisms — Polypharmacy / fall-risk-increasing drugs; balance dysfunction	Polypharmacy; inappropriate drug use; postural stability; fall history; Short Physical Performance Battery; Activities-specific Balance Confidence Scale; fall risk	Polypharmacy was associated with worse postural-stability indices and lower limits of stability. Polypharmacy and inappropriate drug use were also more frequent among participants with a fall history.
Machado-Duque et al., 2024	Colombia / four clinics	Case-control study	To determine associations between fall-risk-increasing drugs, anticholinergic burden, and in-hospital falls among older hospitalized adults.	n = 1,250; cases n = 250, controls n = 1,000	Mean age 77.4 ± 7.4 years	Male 64.0%; female 36.0%	Shared / multimorbidity-related mechanisms — Polypharmacy / fall-risk-increasing drugs; medication-related adverse effects	In-hospital falls; fall-risk-increasing drugs; anticholinergic risk score; polypharmacy; calcium channel blockers; SGLT2 inhibitors; antipsychotics; NSAIDs	Polypharmacy, calcium-channel blockers, SGLT2 inhibitors, anticonvulsants, antipsychotics, NSAIDs, and higher anticholinergic burden were associated with in-hospital falls among older adults.
Zia et al., 2025	Malaysia / teaching	Case-control	To examine whether potentially	n = 428; fallers n = 263	Fallers 75.3 ± 7.3	Fallers: male 84,	Shared / multimorbidity-related	Falls; recurrent falls; medication	Potentially inappropriate prescriptions were more common

	hospital in greater Kuala Lumpur; setting needs manual confirmation	study	inappropriate prescriptions were associated with falls among community-dwelling older adults.	and non-fallers n = 165	years; non-fallers 72.13 ± 5.5 years; eligibility 65–99 years	female 179; non-fallers: male 55, female 110	mechanisms — Polypharmacy / fall-risk-increasing drugs; potentially inappropriate medication exposure	exposure; potentially inappropriate prescriptions; Beers criteria; STOPP criteria; polypharmacy; comorbidities	among fallers than non-fallers. Medication-related risk remained clinically relevant, although the exact associations varied by screening criteria and adjustment model.
Park et al., 2019	Japan / 58 nursing homes	Case-control study	To investigate fall risk factors and medication classes associated with falls among Japanese nursing-home residents.	n = 1,832; cases n = 916 and matched controls n = 916	≥65 years	Female 75%; male 25%	Shared / multimorbidity-related mechanisms — polypharmacy / fall-risk-increasing drugs; medication-related adverse effects	Falls; medication exposure; current prescriptions; antithrombotics; NSAIDs; antiparkinson drugs; muscle relaxants; antiepileptics; antipsychotics; antidepressants; opioids	Falls were associated with functional dependence, visual impairment, insomnia, dementia, and multiple medication classes. Nervous-system drugs, muscle relaxants, opioids, antidepressants, and related drugs were linked with increased fall risk.
Montero-Odasso et al., 2019	Canada / Gait and Brain Study, London, Ontario	Prospective cohort study	To examine whether polypharmacy was associated with gait performance, gait decline, and falls, and whether gait mediated the medication-fall association.	n = 249	76.6 ± 8.6 years; eligibility ≥65 years	Female 156 (63%); male 93 (37%)	Shared / multimorbidity-related mechanisms — Polypharmacy / fall-risk-increasing drugs; gait impairment	Falls; fall incidence; gait speed; gait variability; stride length; step length; step width; polypharmacy	Polypharmacy was associated with poorer baseline gait, later gait decline, and higher fall incidence. Spatial gait parameters partly mediated the association between medication number and falls.
Deguchi et al., 2019	Japan / community pharmacy outpatient setting	Cross-sectional study	To assess risk factors associated with decreased gait speed among older outpatients with polypharmacy.	n = 31	≥65 years	Male n = 13; female n = 18	Shared / multimorbidity-related mechanisms — Polypharmacy; gait speed decline; medication exposure	Gait speed; polypharmacy; number of medications; calcium channel blockers; lower-limb muscle strength	Number of medications was associated with decreased gait speed. Calcium-channel blocker users also had slower gait speed than non-users, suggesting medication burden and prescription content as fall-related mobility indicators.
Al-Azayzih et al., 2024	Jordan / King Abdullah University Hospital outpatient clinics	Retrospective observational study	To assess prescribing patterns of medications associated with geriatric syndromes and identify factors increasing MAGS prescribing in older adults with multiple comorbidities.	n = 1,087	Median age 71 years (IQR 68–77)	Female 52.7%; male 47.3%	Shared / multimorbidity-related mechanisms — Excessive polypharmacy; fall-risk-increasing drugs; medication-related geriatric syndromes	Polypharmacy; excessive polypharmacy; medication-associated geriatric syndromes; falls; urinary incontinence; depression; antihypertensives; antidiabetics; analgesics	Excessive polypharmacy was highly prevalent and associated with higher MAGS scores. Antihypertensives, analgesics, and antidiabetics were frequent MAGS categories linked to falls, urinary incontinence, and depression.
Huberty et al., 2023	Multinational / DO-HEALTH trial: Switzerland, Germany, Austria, France, Portugal	Secondary analysis of trial data	To examine associations of baseline number of chronic diseases and multimorbidity with all and injurious falls over 3 years in European community-dwelling older adults.	n = 2,155 analysed; original trial sample n = 2,157	Mean 74.9 years; eligibility ≥70 years	Female 62%; male 38%	Shared / multimorbidity-related mechanisms — Multimorbidity	All falls; injurious falls; multimorbidity; number of chronic diseases; fall rate	Each additional chronic disease was associated with higher incidence rates of all and injurious falls. Baseline multimorbidity was also associated with increased incidence of both all falls and injurious falls.
Huang et al., 2026	China / China Health and Retirement Longitudinal Study (CHARLS), 2013–2018	Longitudinal cohort study / secondary analysis of cohort data	To examine the longitudinal association between chronic disease count and fall risk and test whether activity limitations mediated this relationship.	n = 2,400	Mean age around 61 years; eligibility ≥45 years	Female 52.49%; male 47.51% across observations	Shared / multimorbidity-related mechanisms — Multimorbidity; activity limitation; physical function decline	Falls; chronic disease count; multimorbidity; activity limitations; ADL / functional limitations	Chronic disease count showed a linear longitudinal association with fall risk. Activity limitations partially mediated this association, accounting for about one fifth of the relationship.
Yan et al., 2022	China / China Health and Retirement Longitudinal Study (CHARLS), 2011–2018	Longitudinal cohort study	To examine longitudinal associations between falls, multimorbidity, and multimorbidity patterns in middle-aged and older adults.	n = 10,015	≥45 years; 45–65 years n = 8,107 (80.9%), ≥66 years n = 1,908 (19.1%)	Female n = 5,390 (53.8%); male n = 4,625 (46.2%)	Shared / multimorbidity-related mechanisms — Multimorbidity; chronic disease patterns	Falls; multimorbidity; chronic disease count; cardiac-metabolic pattern; visceral-arthritic pattern; respiratory pattern; mental-sensory pattern	The number of chronic diseases was positively associated with fall risk. Four multimorbidity patterns were identified, and all were associated with increased falls.
Smith et al., 2025	Multinational / WHO Study on Global Ageing and Adult Health: six low- and middle-income countries	Cross-sectional study	To assess the association between physical multimorbidity and fall-related injury among adults aged ≥50 years in low- and middle-income countries.	n = 34,129	Mean age 62.4 ± 16.0 years; eligibility ≥50 years	Male 48.0%; female 52.0%	Shared / multimorbidity-related mechanisms — multimorbidity; pain burden; mobility limitation; sleep disturbance; cognitive impairment	Fall-related injury; multimorbidity; chronic physical conditions; pain/discomfort; mobility; sleep/energy; cognition	Having two or more chronic conditions was associated with higher odds of fall-related injury. Pain/discomfort, mobility, sleep/energy, and cognition partly mediated the multimorbidity–injury association.
Hoey et al., 2025	Australia / Busselton Healthy Ageing Study	Prospective cohort study	To evaluate associations between multimorbidity count/classes and incident fall- and fracture-related hospitalisations in the Busselton Healthy Ageing Study.	n = 4,991	Age range 45–69 years	Sex/gender NR in extracted text	Shared / multimorbidity-related mechanisms — multimorbidity; cardiometabolic patterns; musculoskeletal patterns	Fall-related hospitalization; fracture-related hospitalization; multimorbidity count; multimorbidity patterns; Cox regression	Each additional morbidity was associated with increased fall-related hospitalisation risk. Cardiometabolic and mental-health/musculoskeletal multimorbidity classes showed higher fall-related hospitalisation risk than the relatively healthy class.
Kim et al., 2022	South Korea / 2017 National Survey of Older Koreans	Cross-sectional study	To identify factors associated with recurrent falls in community-dwelling young-old and old-old adults in South Korea.	n = 10,043; young-old n = 5,838, old-old n = 4,205	≥65 years; young-old 65–74 years, old-old ≥75 years	NR	Shared / multimorbidity-related mechanisms — Multimorbidity; polypharmacy; ADL decline; cognitive impairment	Recurrent falls; chronic disease count; prescribed medications; ADL/IADL; cognitive function; depression; visual aid use; Parkinson’s disease	Factors associated with recurrent falls differed by age group. Chronic disease count and functional limitations were important in both groups, while medication count was significant in the young-old group.
Yang et al., 2023	China / Shaanxi Province; 10 hospitals, 6 community healthcare centers, and 12 village clinics	Cross-sectional study	To identify multimorbidity patterns and examine their associations with gait, balance, and lower-extremity muscle function in older adults.	n = 4,803	Mean age 69.94 ± 6.68 years; eligibility ≥60 years	Male n = 2,276; female n = 2,527	Shared / multimorbidity-related mechanisms — Multimorbidity; gait impairment; balance dysfunction; muscle weakness	Gait speed; Timed Up and Go; 5-times sit-to-stand; multimorbidity patterns; balance performance; lower-extremity muscle function	Five multimorbidity patterns were identified. Degenerative disease and stroke–respiratory–depression patterns were closely associated with gait, balance, and lower-extremity muscle-function impairments, major intrinsic fall-risk factors.
Tchalla et al., 2023	France / FREEDOM Cohort Study	Longitudinal cohort study	To identify frailty trajectory subgroups, baseline predictors of those trajectories, and associated	n = 497	>75 years, or >65 years with at least two comorbidities; mean	NR	Shared / multimorbidity-related mechanisms — Frailty; cognitive impairment; depression / psychological	Frailty; falls; hospitalization; ADL; cognitive function; depression	Frailty trajectories were associated with clinical outcomes, including falls and hospitalization. Poorer trajectories were predicted by older age, cognitive deficit or dementia,

			clinical outcomes in community-dwelling older adults.		age NR in extracted text		symptoms		depressive symptoms, and hypertension.
Welsh et al., 2019	United Kingdom / North Staffordshire Osteoarthritis Project (NorStOP) linked with primary and secondary healthcare records	Prospective cohort study	To examine whether increasing number of pain sites and widespread pain predicted future self-reported falls and falls requiring healthcare use in older adults.	n = 11,375 baseline sample; six-year follow-up n = 4,386	≥50 years	Male n = 5,263; female n = 6,112	Shared / multimorbidity-related mechanisms — Pain burden; multisite pain; physical function decline	Self-reported falls; falls requiring primary healthcare; falls requiring hospital admission; number of pain sites; widespread pain; dizziness; cognitive complaint; medication exposure	Increasing pain-site count predicted three- and six-year self-reported falls. Widespread pain predicted six-year self-reported falls and primary-care fall records, but multisite pain was not clearly associated with hospital-admission falls after adjustment.
Rundell et al., 2021	United States / National Health and Aging Trends Study	Retrospective cohort study / secondary analysis of longitudinal cohort data	To determine whether number and type of chronic conditions were associated with physical performance and recurrent falls over time among older adults with back pain.	n = 2,438	≥65 years; mostly 65–74 years	Female 62%; male 38%	Shared / multimorbidity-related mechanisms — multimorbidity; pain burden; physical function decline; depression / psychological symptoms	Recurrent falls; Short Physical Performance Battery; back pain; chronic conditions; arthritis; depression; anxiety; physical performance	Multiple chronic conditions were associated with poorer SPPB performance and higher odds of recurrent falls among older adults with back pain. Arthritis, depression, and anxiety combinations showed additional physical-performance burden.
Hirase et al., 2020	Australia / eastern Sydney community-dwelling older adults	Prospective cohort study	To investigate whether pain was associated with reactive balance impairment and prospective fall outcomes in community-dwelling older adults.	n = 242	Mean age 80.0 ± 4.4 years; eligibility ≥70 years	Male n = 132; female n = 110	Shared / multimorbidity-related mechanisms — Pain burden; balance dysfunction; recurrent falls	Pain; reactive balance; force-controlled waist-pull perturbation; step initiation time; step velocity; step length; multiple falls; falls with fracture	Pain was associated with poorer reactive balance and increased risk of multiple falls. Reactive balance partially mediated the association between back/neck pain and fall-related fractures.
Gálvez-Barrón et al., 2020	Spain / non-institutionalized older adults sampled nationally	Prospective cohort study	To prospectively evaluate whether pain intensity was associated with recurrent falls in older and oldest-old non-institutionalized adults.	n = 772 enrolled; 630 completed 12-month follow-up; older subgroup n = 222, oldest-old subgroup n = 550	≥65 years; subgroups 65–79 years and ≥80 years	Overall exact sex distribution NR; sex reported by age and pain-intensity strata	Shared / multimorbidity-related mechanisms — Pain burden; recurrent falls; balance and muscle strength	Pain intensity; Face Pain Scale; recurrent falls; balance performance; muscle strength; cognitive decline; depression; medication exposure	Pain intensity was associated with recurrent falls among adults aged 65–79 years after adjustment, but this association was not demonstrated in the ≥80-year subgroup.
Tse et al., 2022	Australia / ASPREE Longitudinal Study of Older Persons (ALSOP)	Longitudinal cohort study	To examine whether low back or lower limb pain severity was associated with recurrent falls and fall-related injuries in older Australians.	n = 14,892; recurrent fallers n = 1,983	≥70 years	Males n = 5,133; females n = 5,377 in recurrent-fall table subset; exact full sample sex NR	Shared / multimorbidity-related mechanisms — Pain burden; recurrent falls; physical function decline	Recurrent falls; fall-related injuries; low back pain; lower limb pain; pain severity; gait speed; grip strength; walking aid use	Severe low back or lower limb pain was associated with recurrent falls in older men and women. Severe pain was also associated with fall-related injury among female recurrent fallers.
Alrawaili, 2024	United States / National Social Life, Health, and Aging Project dataset	Cross-sectional study	To examine independent associations of multisite pain and pain intensity with fall history and frequent falls in community-dwelling older adults.	n = 3,196	≥60 years	NR	Shared / multimorbidity-related mechanisms — Pain burden; multisite pain; medication exposure; multimorbidity	Falls; fall history; frequent falls; multisite pain; pain intensity; medications; comorbidities	Multisite pain and higher pain intensity were associated with fall history after adjustment for demographic and clinical factors. Findings support pain burden as a fall-related mechanism.
Hirase et al., 2020	Japan; first-author affiliation	Systematic review and meta-analysis	To systematically review and synthesize evidence on the impact of pain on static, dynamic, multicomponent, and reactive balance in community-dwelling older adults.	39 studies; total participants n = 17,626	Minimum age ≥60 years	NR	Shared / multimorbidity-related mechanisms — Pain burden; balance dysfunction	Pain; static balance; dynamic balance; multicomponent balance; reactive balance; fall risk	Pain was associated with poorer balance across static, dynamic, multicomponent, and reactive modalities. Chronic pain showed a larger detrimental balance effect than pain of unspecified duration.
Seydi et al., 2024	Australia; first-author affiliation	Systematic review and meta-analysis	To systematically review and meta-analyze the association between pain and objective gait characteristics in older adults.	14 studies; total participants NR	≥60 years	Participant-level sex/gender NR	Shared / multimorbidity-related mechanisms — pain burden; gait impairment; physical function decline	Pain; gait speed; cadence; stride length; double-limb support time; mobility limitation; fall risk	Older adults with pain had significantly slower gait speed than those without pain, with small effect size. Cadence, stride length, and double-limb support time were not consistently different.
Öztürk & Polat, 2026	Turkey / Burdur State Hospital physical therapy unit	Cross-sectional study	To investigate the interrelationship between pain, kinesiophobia, and fear of falling in geriatric patients.	n = 100	Mean age 70.6 ± 4.5 years; eligibility ≥65 years	Sex/gender NR in extracted text	Shared / multimorbidity-related mechanisms — pain burden; kinesiophobia / fear of movement; fear of falling	Pain intensity; Verbal Category Scale; Tampa Kinesiophobia Scale; Tinetti Falls Efficacy Scale; fall history; medication use; chronic diseases	Fear of movement and fear of falling were strongly correlated, while pain showed associations with selected subgroup differences. The cross-sectional design limits causal interpretation of pain–kinesiophobia–fall relationships.
Kitayuguchi et al., 2017	Japan / community-dwelling older adults	Prospective cohort study	To examine whether chronicity and intensity of low back pain or knee pain were associated with falls among community-dwelling older adults.	n = 1,890 analytical sample; baseline responders n = 2,534	Age range 60–79 years	NR	Shared / multimorbidity-related mechanisms — Pain burden; recurrent falls; injurious falls	Falls; multiple falls; injurious falls; low back pain; knee pain; chronic pain; pain intensity	Low back pain chronicity and intensity were associated with injurious falls, and knee pain was associated with at least one fall. Persistently chronic pain was linked to greater fall risk.
Guerreiro et al., 2022	Portugal / Algarve region community-living older adults	Cross-sectional study	To assess mobility and fall risk in community-living older people and identify reliable independent measures predicting mobility loss and fall risk.	n = 192	Mean age 77.93 ± 8.38 years; range 61–101 years	Female n = 141; male n = 51	Shared / multimorbidity-related mechanisms — Pain burden; physical function decline; general falls epidemiology	Fall risk; Tinetti Test; Modified Falls Efficacy Scale; EASY-Care; mobility; pain; osteoarthritis; female sex; ADL	Pain, osteoarthritis, and female sex were identified as relevant characteristics in older people with fall risk. The combined assessment profile better predicted fall risk than isolated categories.
Jacob et al., 2022	Ireland / The Irish Longitudinal Study on Ageing (TILDA)	Prospective cohort study	To analyze the prospective association between multimorbidity and falls and	n = 6,900	Mean age 63.1 ± 8.9 years; eligibility ≥50 years	Female 51.6%; male 48.4%	Shared / multimorbidity-related mechanisms — Multimorbidity; polypharmacy; pain burden; ADL decline;	Falls; multimorbidity; pain; polypharmacy; ADL limitation; sleep problems; cognitive impairment;	Multimorbidity was prospectively associated with falls. Pain, polypharmacy, ADL difficulty, sleep problems, and cognitive impairment contributed as mediating pathways.

			identify mediators of this relationship.				sleep disturbance; cognitive impairment	handgrip strength; obesity	
Lipsitz et al., 2018	United States / MOBILIZE Boston Study	Prospective cohort study	To determine prevalence and progression of peripheral sensory deficits in older adults and whether sensory change was associated with gait-speed decline and falls.	n = 351	Older adults; exact age NR in extracted text	Sex/gender NR in extracted text	Shared / multimorbidity-related mechanisms — peripheral sensory loss; gait impairment; balance dysfunction; neuropathy-related mechanism	Peripheral sensory loss; gait speed; falls; Berg Balance Scale; monofilament sensation; sensory impairment progression	Progressive or persistent peripheral sensory impairment was associated with greater gait-speed decline and increased fall risk. Participants progressing to impairment had higher adjusted fall risk than those with intact sensation.
Canever et al., 2022	Brazil / Balneário Arroio do Silva, Santa Catarina	Cross-sectional study	To investigate whether multimorbidity patterns were associated with fear of falling in community-dwelling older adults.	n = 308	≥60 years; exact mean age NR in extracted text	NR	Shared / multimorbidity-related mechanisms — Multimorbidity; fear of falling	Fear of falling; Falls Efficacy Scale-International; multimorbidity patterns; cardiopulmonary pattern; musculoskeletal pattern; vascular-metabolic pattern	Cardiopulmonary, musculoskeletal, vascular-metabolic, and combined multimorbidity patterns were each associated with higher odds of fear of falling.
Jumani & Powell, 2017	United Kingdom / Sunderland Royal Hospital multidisciplinary balance unit	Retrospective observational study	To assess whether management of benign paroxysmal positional vertigo was associated with reduced falls in older patients referred primarily for falls.	n = 40 fall-referred older adults with BPPV; 873 total BPPV patients screened	65–95 years	Female n = 27; male n = 13	Shared / multimorbidity-related mechanisms — Dizziness / vestibular symptoms; balance dysfunction	Falls; fall frequency; BPPV; Dix-Hallpike test; canalith repositioning treatment; dizziness; comorbidities	Falls decreased from 128 before treatment to 46 after BPPV management. Thirty-eight of 40 patients showed reduced fall incidence at 6-month follow-up, suggesting a vestibular fall-related pathway.
Lin et al., 2023	China / community-dwelling older adults in Guangzhou	Cross-sectional study	To estimate the prevalence of falls and fall injuries and examine associations with chronic diseases among community-dwelling older adults in Guangzhou.	n = 1,629	≥65 years	Female 62.31%; male 37.69%	Shared / multimorbidity-related mechanisms — Multimorbidity; chronic disease burden; general falls epidemiology	Falls; injury from falls; hypertension; diabetes; chronic respiratory disease; chronic disease count; lifestyle factors	Falls were reported by 15.41% and fall injuries by 9.46% of participants. Chronic disease burden and specific chronic diseases were associated with falls and fall-related injuries.
Bai et al., 2026	China / China Health and Retirement Longitudinal Study (CHARLS), 2011–2018	Secondary analysis of cohort data	To examine whether sleep duration was longitudinally associated with fall risk and whether physical function, chronic disease burden, cognition, and IADL mediated this association.	n = 11,476	Mean age 58.81 ± 9.13 years; eligibility ≥45 years	Male 5,621; female 5,855	Shared / multimorbidity-related mechanisms — Sleep disturbance; physical function decline; chronic disease burden; cognitive impairment; IADL decline	Falls; sleep duration; physical function; chronic disease burden; cognitive function; IADL; pain; depressive symptoms; sarcopenia	Short sleep duration was associated with higher future fall risk. The association was partly mediated by physical function, chronic disease burden, cognitive function, and IADL, supporting sleep-related multimorbidity pathways.
Zhang et al., 2022	United States / National Health and Nutrition Examination Survey (NHANES), 2005–2008	Cross-sectional study	To examine whether self-reported sleep duration and sleep characteristics were associated with falls-associated fractures in older adults.	n = 4,321 with complete data; falls-associated fracture group n = 168 and control group n = 4,153	Falls-associated fracture group 72.1 ± 8.8 years; control group 62.0 ± 11.2 years; eligibility ≥45 years	Falls-associated fracture group: male 55, female 113; control group: male 2,190, female 1,963	Shared / multimorbidity-related mechanisms — Sleep disturbance; osteoporosis/fracture-related fall outcome	Falls-associated fractures; sleep duration; snoring; hypertension; osteoporosis; living arrangement	Very short or long sleep duration was associated with higher odds of falls-associated fractures compared with 6–8 hours of sleep. Older age, sex, and osteoporosis also differed between fracture and control groups.
Donovan et al., 2023	Australia; first-author affiliation	Systematic review and meta-analysis	To estimate the prevalence of vestibular dysfunction and associated factors among people who fall.	10 studies; total participants n = 468	Mean age across included studies ranged from 62 to 82 years	Female proportion across included studies ranged from 68% to 95%	Shared / multimorbidity-related mechanisms — Vestibular dysfunction; dizziness; balance dysfunction	Falls; vestibular dysfunction; BPPV; vestibulo-ocular reflex dysfunction; walking function; dizziness; balance confidence	Vestibular dysfunction was common among people who fall, with vestibulo-ocular reflex dysfunction most prevalent. Fallers with vestibular dysfunction had reduced walking function compared with fallers without vestibular dysfunction.
Kwon et al., 2022	South Korea / tertiary dizziness clinic	Retrospective observational study	To describe clinical characteristics and mechanisms of vestibular syncope.	n = 53	Median age 63 years (IQR 54–71)	Female 33; male 20	HTN-related mechanisms — Dizziness / syncope; orthostatic hypotension; baroreflex / vestibulo-sympathetic reflex mechanisms	Vestibular syncope; recurrent syncope; falls; injuries; orthostatic hypotension; vestibular function; hypertension; baroreflex function	Vestibular syncope was recurrent in 37.7% of patients and caused serious injuries in 13.2%. The proposed mechanism involved vestibulo-sympathetic and baroreflex pathways, with orthostatic hypotension present in a subset.
Dai et al., 2018	Singapore / Singapore Epidemiology of Eye Diseases Study	Cross-sectional population-based study	To examine prevalence and risk factors for falls and recurrent falls in a multi-ethnic Asian adult population.	n = 10,009	Mean age 58.9 ± 10.4 years	NR	Shared / multimorbidity-related mechanisms — General falls epidemiology; multimorbidity; visual impairment; diabetes and cardiovascular disease	Falls; recurrent falls; diabetes; cardiovascular disease; multimorbidity; visual impairment; EQ-5D; quality of life	Older age, female sex, diabetes, cardiovascular disease, multimorbidity, visual impairment, and poorer health-related quality of life were associated with falls or recurrent falls.
Rivasi et al., 2023	Italy; first-author affiliation	Narrative review	To review risks associated with intensive blood pressure control in older patients, including hypotension, syncope, falls, and other adverse outcomes.	N/A; narrative review	Older adults; exact age NR	N/A; narrative review	HTN-related mechanisms — Low systolic blood pressure; treatment intensity; antihypertensive medication exposure; dizziness / syncope; falls	Blood pressure control; hypotension; syncope; falls; antihypertensive treatment; renal outcomes; cognitive function; quality of life; mortality	The review summarized evidence that intensive blood-pressure control may benefit selected older adults but can increase safety concerns in frail or multimorbid patients, including hypotension, syncope, and fall-related outcomes.
Fan et al., 2024	China / Southern Medical University and related rehabilitation laboratory settings	Cross-sectional experimental study	To compare center-of-pressure parameters and cortical activation during standing balance tasks in older adults with hypertension versus normotension.	n = 57; normotension n = 30, hypertension n = 27	NT group 69.30 ± 3.16 years; HT group 70.30 ± 4.07 years	Female n = 30; male n = 27	HTN-related mechanisms — Cerebral oxygenation; balance dysfunction; blood-pressure-related neural control	Center of pressure; standing balance; fNIRS; cortical oxygenation; Berg Balance Scale; Activities-specific Balance Confidence Scale; fall history	Standing balance performance did not differ clearly across most COP parameters, but hypertensive older adults showed lower activation in the premotor/supplementary motor cortex during the most difficult balance task.
Wang et al., 2022	China / community-dwelling older adults in Pudong New Area, Shanghai	Cross-sectional study	To examine whether cognitive frailty was associated with falls among community-dwelling older adults with hypertension.	n = 305	Age range 60–89 years	Female n = 197; male n = 108	HTN-related mechanisms — Cognitive impairment; frailty; multiple shared mechanisms	Falls; cognitive frailty; physical frailty; mild cognitive impairment; depression; hypertension	Cognitive frailty was associated with falls among older adults with hypertension. Other fall-related factors included older age, female sex, hearing impairment, depression, and greater prescription-medication exposure.
Abu Bakar et al.,	Malaysia / two	Cross-sectional	To determine the prevalence of	n = 269	Mean age 71.00 ± 5.24	Female n = 160;	HTN-related mechanisms —	Falls; hypertension; polypharmacy;	Falls were reported by 32.2% of hypertensive older adults.

2021	primary health clinics in Kuala Terengganu	study	falls and identify associated factors among older adults with hypertension.		years; eligibility ≥65 years	male n = 109	Antihypertensive medication exposure; diuretics / medication class; balance dysfunction	diuretics; Performance Oriented Mobility Assessment; visual impairment; Barthel Index; antihypertensive medication	Polypharmacy and diuretic use were independently associated with increased fall risk, whereas number of antihypertensives was not clearly associated.
Toba et al., 2019	Japan / outpatient clinic	Cross-sectional study	To investigate the relationship between orthostatic blood-pressure rise and frailty in older outpatients.	n = 120	Mean age 77.4 years	NR	HTN-related mechanisms — Orthostatic blood pressure change; frailty; multiple hemodynamic mechanisms	Orthostatic blood pressure; frailty; gait speed; Timed Up and Go; grip strength; one-leg standing; falls; fear of falling	Orthostatic blood-pressure rise was associated with frailty in older patients. The study suggested postural BP dysregulation as a geriatric vulnerability marker relevant to mobility and fall-related outcomes.
Teng et al., 2023	China / community-dwelling older adults in Wuxi	Cross-sectional study	To examine the relationship between frailty status, hypertension, and fall risk in community-dwelling older adults.	n = 401	≥60 years; age groups reported	Men 154 (38.4%); women 247 (61.6%)	HTN-related mechanisms — Frailty; hypertension-related shared fall-risk mechanisms	Fall risk; STEADI fall-risk checklist; frailty; hypertension; comorbidities; polypharmacy	Frailty and hypertension were independently associated with higher fall risk. Older adults with both frailty and hypertension had the highest fall-risk prevalence, and frailty showed a nonlinear association with fall risk by hypertension status.
Sim et al., 2018	United States / Kaiser Permanente Southern California	Cross-sectional study	To evaluate whether low treated systolic blood pressure was associated with serious falls and syncope among treated hypertensive adults.	n = 477,516 treated hypertensive individuals	Mean 65 ± 13 years	NR	HTN-related mechanisms — Low systolic blood pressure; antihypertensive medication exposure; dizziness / syncope	Serious falls; syncope; minimum systolic blood pressure; mean systolic blood pressure; medication exposure	Minimum or mean treated SBP below 110 mmHg was associated with higher odds of serious falls and syncope among treated hypertensive patients. Findings support low-BP safety monitoring during intensive BP management.
Stama et al., 2025	Netherlands / Leiden University Medical Centre tilt-table-test database	Retrospective observational study	To examine hemodynamic determinants of supine hypertension in patients with classical orthostatic hypotension using tilt-table-test data.	n = 160; cOH n = 80, controls n = 80	Controls median 65 years (range 50–87); cOH median 68 years (range 43–90)	Female n = 50; male n = 110	HTN-related mechanisms — Orthostatic hypotension; supine hypertension; blood pressure dysregulation; cerebral hypoperfusion context	Classical orthostatic hypotension; supine hypertension; systolic blood pressure; diastolic blood pressure; heart rate; stroke volume; total peripheral resistance	Patients with classical orthostatic hypotension and supine hypertension showed distinct hemodynamic profiles. The study maps BP dysregulation mechanisms relevant to orthostatic symptoms, syncope, and fall vulnerability.
Wang et al., 2025	China / Chinese Longitudinal Healthy Longevity Survey (CLHLS) 2018	Cross-sectional study	To investigate factors associated with falls and gender differences among older Chinese adults with hypertension.	n = 2,814 analytical sample; CLHLS 2018 surveyed n = 15,874	≥65 years	Female n = 1,670; male n = 1,144	HTN-related mechanisms — Multiple hemodynamic / comorbidity-related mechanisms; ADL decline; stroke; sensory impairment	Falls; hypertension; self-rated health; hearing impairment; stroke; BADL; IADL; exercise; fruit intake; taste preference	Falls occurred in 22.60% of older adults with hypertension. Fall-associated factors included sex, self-rated health, hearing impairment, stroke, BADL/IADL disability, exercise, fruit intake, and taste preference, with gender-specific patterns.
Klop et al., 2025	Netherlands / Radboudumc and Amstelland Hospital plus community-recruited groups	Experimental / physiological laboratory study	To determine whether age, sex, antihypertensive use, comorbidity, and orthostatic hypotension were associated with baroreflex sensitivity and cerebral oxygenation after postural change.	n = 105; younger adults n = 34, community-recruited older adults n = 30, geriatric outpatients n = 41	Younger adults median 25 years; older adults median 77 years; geriatric outpatients median 76 years	Female: younger adults 10/34 (29%); older adults 17/30 (55%); geriatric outpatients 19/41 (46%)	HTN-related mechanisms — Orthostatic hypotension; cerebral hypoperfusion / cerebral oxygenation; baroreflex sensitivity	Orthostatic hypotension; baroreflex sensitivity; cerebral oxygenation recovery; continuous blood pressure; ECG; near-infrared spectroscopy; cerebral autoregulation	Higher age and orthostatic hypotension were associated with lower baroreflex sensitivity. Orthostatic hypotension was also associated with reduced cerebral oxygenation recovery after postural change, while clear cerebral-autoregulation impairment was not found.
Bourke et al., 2024	Ireland; first-author affiliation	Systematic review and meta-analysis	To systematically review and meta-analyze evidence on associations between cardiovascular disorders and falls in adults aged 50 years and older.	184 studies; 181 observational and 3 interventional	≥50 years	N/A; systematic review	HTN-related mechanisms — Orthostatic hypotension; syncope; arrhythmia; multiple cardiovascular / hemodynamic mechanisms	Falls; orthostatic hypotension; hypertension; arrhythmia; valvular disease; carotid sinus hypersensitivity; cardiovascular interventions	The review found several cardiovascular disorders, including orthostatic hypotension, arrhythmia, valvular disease, and carotid sinus hypersensitivity, were associated with falls. Evidence was stronger for some measurement methods than others.
Dani et al., 2021	United Kingdom; first-author affiliation	Narrative review	To review diagnosis and management considerations for orthostatic hypotension in older people, including falls-related consequences.	N/A; narrative review	Older adults; exact age NR	N/A; narrative review	HTN-related mechanisms — Orthostatic hypotension; cerebral hypoperfusion / cerebral oxygenation; dizziness / syncope	Orthostatic blood pressure; dizziness; syncope; falls; fractures; supine hypertension	The review described orthostatic hypotension as common in older people and linked to falls, fractures, syncope, dementia, and death. It summarized diagnostic testing and nonpharmacological and medication-based management strategies.
Aubert et al., 2021	United States / Veterans Health Administration and Medicare data	Retrospective cohort study	To assess whether antihypertensive treatment deintensification or intensification was associated with clinical outcomes among older adults treated to low blood pressure.	n = 228,753	Mean age 75 ± 7.5 years; eligibility ≥65 years	Male 98.2%	HTN-related mechanisms — Low systolic blood pressure; antihypertensive treatment intensity; syncope / falls	Fall injury; syncope; systolic blood pressure; antihypertensive medication dose; treatment deintensification; treatment intensification	Compared with stable treatment, antihypertensive deintensification or intensification was not associated with lower fall injury or syncope risk among older adults with tightly controlled SBP.
Margolis et al., 2019	United States / Women's Health Initiative OPACH study	Prospective cohort study	To examine whether hypertension treatment/control status, measured blood pressure, and antihypertensive drug class were associated with one-year fall incidence in older women.	n = 5,971	Mean age 79 years	Female 100%	HTN-related mechanisms — antihypertensive medication exposure; low diastolic blood pressure; medication class	Falls; fall incidence; systolic blood pressure; diastolic blood pressure; antihypertensive medication; beta blockers; Short Physical Performance Battery	Treated controlled and treated uncontrolled hypertension were not associated with higher fall rates versus no hypertension and were associated with lower fall rates after adjustment. Beta blockers were the only antihypertensive class associated with increased falls before SPPB adjustment.
Omer et al., 2018	United Kingdom / University Hospitals Birmingham NHS Trust	Retrospective cohort study	To investigate whether antihypertensive medications were adjusted after inpatient falls among older adults with normal or low blood pressure.	n = 146	≥80 years	Sex/gender NR in extracted text	HTN-related mechanisms — low blood pressure; antihypertensive medication exposure; post-fall medication review	Inpatient falls; antihypertensive prescribing; blood pressure; medication review; dose reduction; discharge medications	Most patients had no antihypertensive reduction within 24 hours after a fall, and new antihypertensives were sometimes started. At discharge, medication changes were more common but many prescriptions remained unchanged.
Aurelian et al., 2025	Romania / geriatric and medical university hospital settings	Observational case-control study	To examine the association between cardiovascular pharmacotherapy and fall risk in older adults and identify	n = 200; high fall-risk group n = 100, low-risk controls n = 100	Eligibility >55 years; exact mean age NR	Female n = 157 (78.5%); male n = 43 (21.5%)	HTN-related mechanisms — Antihypertensive medication exposure; diuretics / beta-blockers / medication class; polypharmacy	Fall risk; Downton Fall Risk Index; systolic blood pressure; cardiovascular medication; beta-blockers; calcium channel blockers; spironolactone; frailty;	High fall-risk participants differed in cardiovascular profile and medication exposure. Spironolactone, beta-blockers, calcium-channel blockers, frailty, cognitive impairment, diabetes, and osteoarticular conditions were associated with

			preventive strategies.					cognitive function	fall risk.
Şirali, 2025	Turkey / Ufuk University Hospital	Retrospective observational study	To evaluate the role of hyponatremia and antihypertensive medications in patients admitted with fall-related injuries.	n = 322	Mean age 73.28 ± 14.26 years	Female 60.2%; male 39.8%	HTN-related mechanisms — electrolyte imbalance / volume depletion; antihypertensive medication exposure; diuretics / beta-blockers / medication class	Fall-related injuries; fractures; serum sodium; hyponatremia; hydrochlorothiazide; ACE inhibitors; beta blockers; ACE+HCT; SSRI use	Hyponatremia was associated with antihypertensive classes including hydrochlorothiazide, ACE inhibitors, beta blockers, and ACE+HCT. Hyponatremia and female sex were associated with fracture occurrence among fall-related injury patients.
Sink et al., 2018	United States and Puerto Rico / SPRINT trial	Randomized controlled trial	To determine predictors of serious adverse events for syncope, hypotension, and falls in SPRINT, including whether intensive blood-pressure treatment increased fall-related harms.	n = 9,361	≥50 years	Sex/gender NR in extracted text	HTN-related mechanisms — intensive blood-pressure treatment; hypotension; syncope; falls	Serious adverse events; falls; syncope; hypotension; systolic blood pressure; frailty; chronic kidney disease	Intensive systolic blood-pressure control increased serious hypotension and possibly syncope, but not serious falls. Older age, frailty, and chronic kidney disease increased event risk, without clear age-by-treatment interaction.
Donoghue et al., 2021	Ireland / The Irish Longitudinal Study on Ageing (TILDA)	Longitudinal cohort study	To estimate whether orthostatic hypotension and co-existing supine or seated hypertension were associated with future recurrent, injurious, unexplained falls and syncope.	n = 1,500	Mean age 71.1 ± 5.2 years	Female n = 765 (51.0%); male n = 735 (49.0%)	HTN-related mechanisms — Orthostatic hypotension; blood pressure variability; dizziness / syncope; hemodynamic instability	Recurrent falls; injurious falls; unexplained falls; syncope; orthostatic blood pressure; supine hypertension; seated hypertension; postural dizziness	Associations were mixed. Sustained orthostatic hypotension with co-existing hypertension was more consistently associated with injurious or unexplained future falls than orthostatic hypotension alone.
Hailu et al., 2024	Ethiopia / University of Gondar Hospital, hypertension clinic	Cross-sectional study	To determine the prevalence of orthostatic hypotension and associated factors among older people with hypertension in northern Ethiopia.	n = 240	Mean age 68.8 ± 7.15 years; range 60–92 years	Male n = 107 (44.8%); female n = 132 (55.2%); n = 239 for sex	HTN-related mechanisms — Orthostatic hypotension; antihypertensive medication exposure	Orthostatic blood pressure; orthostatic hypotension; OH symptoms; antihypertensive medication class; calcium channel blockers	Orthostatic hypotension affected 23.8% of older hypertensive patients. Calcium-channel blocker use was independently associated with OH, and most patients with measured OH reported related symptoms.
Juraschek et al., 2022	United States / STURDY trial	Secondary analysis of randomized trial data	To compare seated-to-standing and supine-to-standing orthostatic hypotension protocols for prevalence, incident falls, and orthostatic symptoms.	n = 534	Mean age 76 ± 5 years; eligibility ≥70 years	Female 42%; male 58%	HTN-related mechanisms — orthostatic hypotension; postural blood-pressure change; dizziness / orthostatic symptoms	Falls; recurrent falls; orthostatic hypotension; seated blood pressure; supine blood pressure; orthostatic symptoms	Supine-to-standing OH was more common than seated-to-standing OH and better captured clinically relevant orthostatic symptoms. Associations with incident falls differed by protocol and were not interchangeable.
Wiersinga et al., 2024	Netherlands; first-author affiliation	Narrative review	To review the pathophysiology, guideline issues, and clinical management of coexisting hypertension and orthostatic hypotension in older adults.	N/A; narrative review	Older adults; exact age NR	N/A; narrative review	HTN-related mechanisms — orthostatic hypotension; blood-pressure dysregulation; dizziness / falls; antihypertensive medication exposure	Hypertension; orthostatic hypotension; falls; frailty; blood pressure regulation; antihypertensive treatment; calcium channel blockers; ACE inhibitors	The review described hypertension and orthostatic hypotension as intertwined conditions in older adults. It emphasized individualized treatment, careful monitoring, and fall-related caution rather than routine avoidance of hypertension treatment.
Magkas et al., 2019	Greece; first-author affiliation	Narrative review	To review orthostatic hypotension definitions, diagnostic approaches, pathophysiology, prognosis, and therapeutic considerations.	N/A; narrative review	Older and frail adults discussed; exact age NR	N/A; narrative review	HTN-related mechanisms — Orthostatic hypotension; cerebral hypoperfusion / cerebral oxygenation; dizziness / syncope	Orthostatic hypotension; blood pressure; syncope; dizziness; cerebral hypoperfusion; falls; antihypertensive treatment	The review described orthostatic hypotension as a hemodynamic and autonomic disorder associated with dizziness, syncope, cerebral hypoperfusion, impaired quality of life, and fall-related vulnerability.
Samajdar et al., 2022	India / urban municipality area of Kolkata, West Bengal	Record-based cross-sectional study	To assess whether polypharmacy and anticholinergic burden were associated with cardiovascular autonomic function among older adults with chronic diseases.	n = 305	Mean age 68.9 ± 3.4 years	Male n = 201 (65.9%); female n = 104 (34.1%)	Shared / multimorbidity-related mechanisms — Polypharmacy / fall-risk-increasing drugs; HTN-related orthostatic hypotension; autonomic dysfunction	Polypharmacy; anticholinergic burden; cardiac autonomic neuropathy; orthostatic hypotension; Valsalva ratio; handgrip test; blood pressure	Polypharmacy and higher anticholinergic burden were associated with cardiac autonomic neuropathy among older adults with chronic diseases, indicating a medication-related autonomic pathway relevant to orthostatic instability.
Cemali et al., 2024	Turkey	Cross-sectional study	To compare sensory processing, kinesiophobia, and fear of falling between older adults with hypertension and normotension.	n = 124; hypertension n = 62, normotension n = 62	65–84 years	NR	HTN-related mechanisms — Vestibular / visual processing; kinesiophobia / fear of movement; fear of falling; balance-related sensory processing	Fear of falling; kinesiophobia; vestibular processing; visual processing; sensory processing; activity level; hypertension status	Older adults with hypertension showed higher fear of falling and kinesiophobia and poorer vestibular, visual, and activity processing than normotensive peers. Age and sensory-processing variables were related to fear-of-falling outcomes.
Naseer et al., 2025	Pakistan / Bajwa Hospital, Lahore	Cross-sectional study	To examine associations among anthropometric measures, postural balance, fear of falling, fall risk awareness, and quality of life in older females by hypertensive status.	n = 114	Age 65–83 years; overall 71.7 ± 5.0 years; hypertensive 73.1 ± 5.8, normotensive 70.3 ± 4.6	Female 114/114 (100%)	HTN-related mechanisms — Balance dysfunction; obesity / anthropometric risk; fear of falling; fall risk awareness	Timed Up and Go; Berg Balance Scale; Falls Efficacy Scale International; fall risk awareness; BMI; waist-to-height ratio; quality of life; blood pressure status	Hypertensive older women had higher anthropometric risk and poorer balance than normotensive women. Balance, fear of falling, and fall-risk awareness predicted selected quality-of-life domains.
Li et al., 2025	China / Huaihe Hospital of Henan University, Department of Geriatrics	Cross-sectional study	To investigate associations of sleep disorders and cognitive frailty with falls among hospitalized older adults with hypertension.	n = 266; fallers n = 108	≥65 years	Sex/gender reported in table; exact overall distribution not extracted clearly	HTN-related mechanisms — Cognitive impairment; sleep disturbance; frailty; polypharmacy; multiple hemodynamic/geriatric mechanisms	Falls; sleep disorders; cognitive frailty; cognitive function; frailty; depression; nutritional status; polypharmacy; comorbidities	Sleep disorders and cognitive frailty were independently associated with falls in hospitalized older adults with hypertension. Age, depression, malnutrition, and cardiovascular disease were also identified as significant fall-related factors.
Thomas et al., 2025	United States / National Health and Aging Trends Study 2021–2022	Retrospective cross-sectional and cohort study	To examine cross-sectional and longitudinal associations between visual impairment and falls among older United States adults.	n = 2,822	≥71 years	NR in current PDF page	Other / background evidence — Vision impairment; general falls epidemiology	Falls; multiple falls; distance visual acuity; near visual acuity; contrast sensitivity; visual impairment	Visual impairment was associated with higher baseline rates of falls and multiple falls. Some longitudinal visual-function changes were associated with subsequent falls, but the article was not matched to the original Excel table.
Mawarikado et al., 2025	Japan	Cross-sectional study	To of this study, therefore, was	n = 51	Older adults	Female 84%; male 16%	OA — Proprioceptive impairment / joint position sense	Falls; Fall risk; Gait parameters; Balance performance; Pain; Proprioception	Fall history in lower-limb OA was related to foot pressure-center parameters during gait and stance, indicating altered balance-control proxies among fallers.

Juraschek et al., 2022	United States / Trial of Nonpharmacologic Interventions in the Elderly (TONE), 4 academic centers in Maryland, New Jersey, North Carolina, and Tennessee	Secondary analysis of trial data	To examine the effects of antihypertensive medication withdrawal and nonpharmacologic replacement strategies on blood pressure, adverse events, and orthostatic symptoms in older adults.	n = 975	Mean age 65.8 ± 4.6 years; eligibility 60–80 years	Female 47.8%; male 52.2%	HTN-related mechanisms — Antihypertensive medication exposure / deprescribing; orthostatic symptoms; blood pressure dysregulation; dizziness / syncope	Systolic blood pressure; diastolic blood pressure; antihypertensive drug withdrawal; orthostatic symptoms; lightheadedness; dizziness; imbalance; adverse events; falls; fracture; syncope	Antihypertensive withdrawal increased SBP and symptomatic adverse events mainly during withdrawal. Combined weight loss/sodium reduction mitigated BP increases and reduced orthostatic symptoms, while sodium reduction alone increased adverse events; interventions were not clearly associated with falls alone.
Korkusuz et al., 2024	Turkey / FizyoCare Physical Therapy and Rehabilitation Center Gait Analysis Laboratory	Prospective controlled laboratory study	To evaluate and compare the effects of neuropathic pain on balance, gait, and plantar pressure distribution in individuals with diabetic peripheral neuropathy.	n = 42; DPN with neuropathic pain n = 14, DPN without neuropathic pain n = 14, control n = 14	DPN+NP 61.71 ± 8.09 years; DPN-NP 63.64 ± 7.42 years; controls 55.86 ± 9.72 years	DPN+NP: female 10 (71.4%), male 4 (28.6%); DPN-NP: female 5 (35.7%), male 9 (64.3%); controls: female 5 (35.7%), male 9 (64.3%)	T2DM-related mechanisms — Neuropathy / sensory-feedback failure; gait impairment; balance dysfunction; foot biomechanics / plantar pressure; neuropathic pain	LANSS; Visual Analog Scale; static balance; dynamic balance; limits of stability; gait parameters; step width; single support line; dynamic plantar pressure; heel maximum force	Both DPN groups showed increased step width, shorter left single-support line, and reduced heel maximum force versus controls. Neuropathic pain was not significantly associated with additional balance or gait differences.
